# CAR-T cell combination therapy: the next revolution in cancer treatment

**DOI:** 10.1186/s12935-022-02778-6

**Published:** 2022-11-24

**Authors:** Maysoon Al-Haideri, Santalia Banne Tondok, Salar Hozhabri Safa, Ali Heidarnejad maleki, Samaneh Rostami, Abduladheem Turki Jalil, Moaed E. Al-Gazally, Fahad Alsaikhan, Jasur Alimdjanovich Rizaev, Talar Ahmad Merza Mohammad, Safa Tahmasebi

**Affiliations:** 1 Department of Physiotherapy, Cihan University, Erbil, Kurdistan Region Iraq; 2 Department of Nursing, Health Polytechnic of the Ministry of Health Jayapura, Jayapura, Indonesia; 3 School of Medicine, Zanjan University of Medical Sciences, Zanjan, Iran; 4 Medical Laboratories Techniques Department, Al-Mustaqbal University College, Hilla, 51001 Babylon Iraq; 5 College of Medicine, University of Al-Ameed, Karbala, Iraq; 6 College of Pharmacy, Prince Sattam Bin Abdulaziz University, Alkharj, Kingdom of Saudi Arabia; 7 Department of Public Health and Healthcare Management, Rector Samarkand State Medical University, Samarkand, Uzbekistan; 8 Department of Pharmacology, College of Pharmacy, Hawler Medical University, Erbil, Kurdistan Region Iraq; 9 Department of Immunology, School of Medicine, Shahid Beheshti University of Medical Sciences, Tehran, Iran

**Keywords:** Chimeric antigen receptor, CAR-T cell therapy, Combination therapy, Hematological malignancies, Solid tumors

## Abstract

In recent decades, the advent of immune-based therapies, most notably Chimeric antigen receptor (CAR)-T cell therapy has revolutionized cancer treatment. The promising results of numerous studies indicate that CAR-T cell therapy has had a remarkable ability and successful performance in treating blood cancers. However, the heterogeneity and immunosuppressive tumor microenvironment (TME) of solid tumors have challenged the effectiveness of these anti-tumor fighters by creating various barriers. Despite the promising results of this therapeutic approach, including tumor degradation and patient improvement, there are some concerns about the efficacy and safety of the widespread use of this treatment in the clinic. Complex and suppressing tumor microenvironment, tumor antigen heterogeneity, the difficulty of cell trafficking, CAR-T cell exhaustion, and reduced cytotoxicity in the tumor site limit the applicability of CAR-T cell therapy and highlights the requiring to improve the performance of this treatment. With this in mind, in the last decade, many efforts have been made to use other treatments for cancer in combination with tuberculosis to increase the effectiveness of CAR-T cell therapy, especially in solid tumors. The combination therapy results have promising consequences for tumor regression and better cancer control compared to single therapies. Therefore, this study aimed to comprehensively discuss different cancer treatment methods in combination with CAR-T cell therapy and their therapeutic outcomes, which can be a helpful perspective for improving cancer treatment in the near future.

## Introduction

Chimeric antigen receptor (CAR)-T cell therapy has emerged to revolutionize cancer treatment, which has achieved promising success, especially in treating hematological malignancies like B-cell acute lymphocytic leukemia (B-ALL), multiple myeloma (MM), and non-Hodgkin lymphoma (NHL). The US Food and Drug Administration (FDA) approved CAR-T cells targeting CD19 and B cell maturation antigen (BCMA) for the treatment of R/R B cell malignancies including tisagenlecleucel (Kymriah), axicabtagene ciloleucel (Yescarta), ciltacabtagene autoleucel (Carvykti), idecabtagene vicleucel (Abecma), brexucabtagene autoleucel (Tecartus), and lisocabtagene maraleucel (Breyanzi). CAR is a chimeric TCR-engineered receptor designed from two parts: an antigen-binding domain composed of a monoclonal antibody and a T-cell receptor (TCR) signaling domain. In detail, CAR is structured in four parts: (1) An extracellular region contains light and heavy fragments of a monoclonal antibody, namely a single-chain variable fragment (ScFv), which involves antigen recognition; (2) A hinge or spacer region links between extra- and- intracellular regions (mostly IgG-derived spacer or CD8), accounting for receptor flexibility and stability; (3) A transmembrane portion contributes to receptor flexibility and mostly needs CD3ζ to dimerization and correlation between other parts of CAR. 4) An intracellular domain composed of the main signaling domain (CD3ζ or FcεRIγ), which may possess one or more costimulatory domains [CD28, 4-1BB (CD137), or OX40 (CD134)], as well [1, 2]. This explained structure provides the specific and non-HLA restricted antigen recognition without requiring antigen processing. Furthermore, the flexible construction of CAR makes it more feasible to recognize the carbohydrate and lipid antigens in addition to peptide antigens [2]. CAR-T cells have been divided into five generations depending on endodomain conformation. Accordingly, the first generation is formed by one signaling domain originating from the TCR/CD3 complex. The absence of costimulatory signals in first-generation CARs elicits poor proliferation, persistence, and anti-tumor cytotoxicity. The second generations is characterized by presenting a costimulatory molecule like CD28, ICOS, OX40, or 4-1BB that aguments their proliferative and cytotoxic capacities compared to first generation. Similar to second generation, the third generations contain two seperate co-stimulatory molecules like CD28 and 4-1BB, which exhibit increased proliferation, cytotoxicity and persistence compered to the two previous generations. The fourth generation CAR, so-called cells redirected for antigen-unrestricted cytokine-initiated killing (TRUCK) or armored CAR, is a kind of structure secreting cytokines [Interluekin (IL)-7, IL-12, IL-15, and IL-21], sucide genes (iCaspase-9) or other biological molecules. TRUCKs act more effecting in inducing the immune responses and eradicating the tumor cells. The fifth generation of CAR is designed based on the structure of the second-generation CAR, which also possesses cytokine receptors in the intracellular domain like of IL-2Rβ chain fragment [3]. Up to now, CAR-T cell therapy has led to promising results and even complete cures for hematological malignancies [4, 5]. However, the success of this treatment in solid tumors has been challenged by the tumor-antigen heterogeneity, impaired CAR-T cell trafficking and infiltration, and the presence of an immunosuppressive tumor microenvironment (TME) [6]. TME is characterized by a physical barrier, inhibitory enzymes, checkpoint inhibitors, immunosuppressive cells, hypoxia, Nutrient Deficiency, and inhibitory cytokines. Proliferation, function, and persistence of CAR-T cells are reduced in the TME with the abovementioned inhibitory structure, which also causes the CAR-T cell anergy and exhaustion [6, 7]. Therefore, targeting different tumor antigens to overcome antigen heterogeneity and increase the CAR-T cell persistence and efficacy using the genetic modifications in CAR structure and combinatorial treatment strategies can help overcome TME hurdles [8, 9]. In addition to the abovementioned barriers, the side effects of CAR-T cell therapy are other significant challenges associated with this kind of treatment. Cytokine storm or cytokine release syndrome (CRS), damage and depletion of bystander healthy cells, so-called “on-target/off-tumor” toxicity, tumor lysis syndrome (TLS), graft versus host disease (GVHD), neurotoxicity, and anaphylactic shock are prevalent complications of CAR-T cell therapy leading to multi-organ failure [10, 11]. For this reason, several therapeutic approaches have been developed so far to increase the safety of CAR-T cell therapy, limit its toxicity, and improve anti-tumor function. This study aimed to comprehensively review and discuss the cancer therapeutic approaches combined with CAR-T cells to intensify the safety and efficacy of cancer CAR-T cell therapy.

## CAR-T Cell combination therapy opportunities

**Fig. 1:**
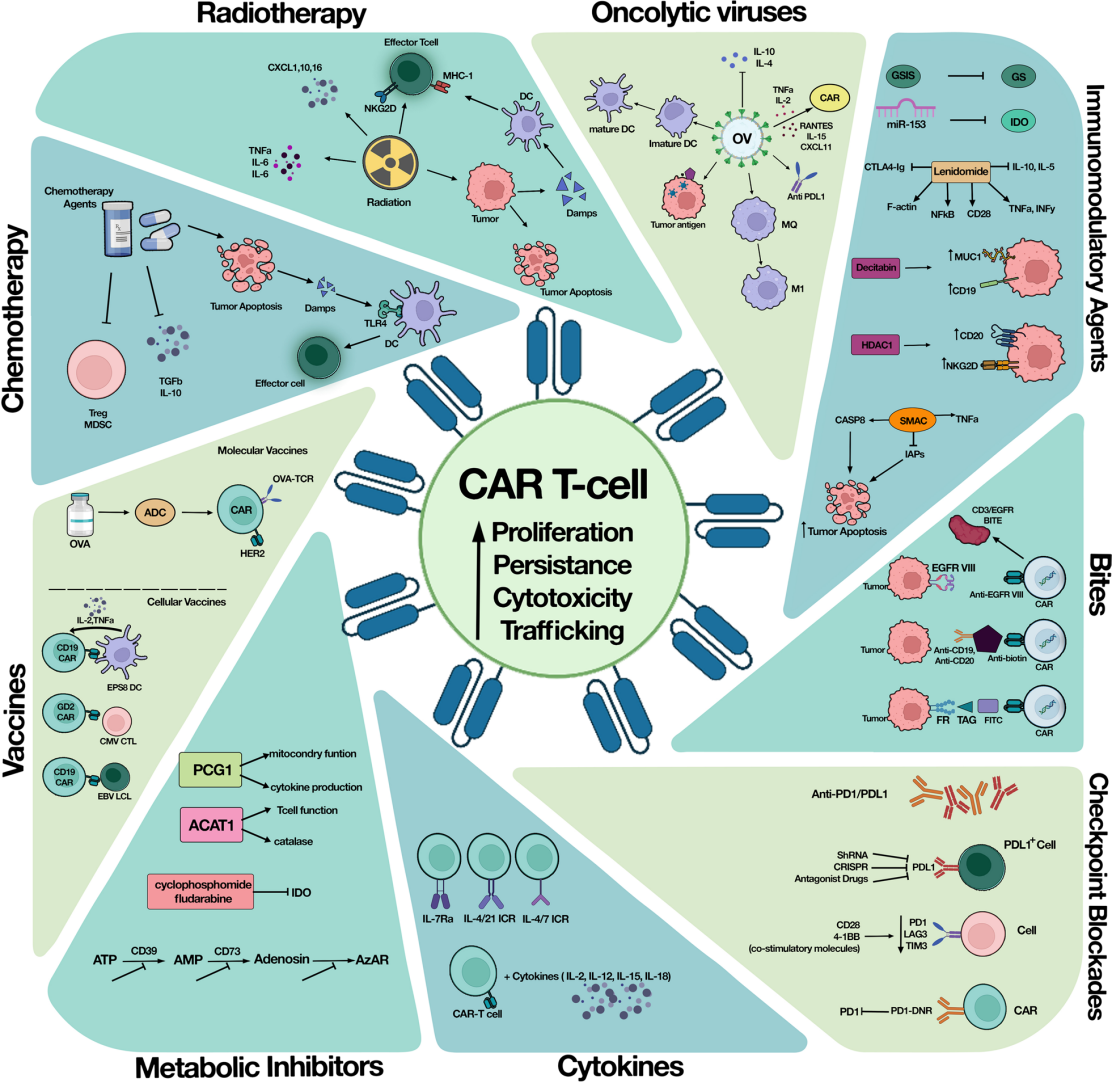
CAR-T cell combination therapy with other cancer therapeutic approaches

To the best of our knowledge, advances in CAR-T cell combination therapy with other therapeutic approaches have opened up promising horizons for more successful cancer treatment, especially for solid tumors. So far, various studies have supported this idea and shown that combination therapy has dramatically increased the effectiveness of CAR-T cell therapy and reduced its side effects [12, 13]. In this case, chemotherapy, radiotherapy, oncolytic viruses, cancer vaccines, cytokines, checkpoint inhibitors, Bi-specific T-cell engagers (BiTEs), immunomodulatory agents, Hematopoietic stem cell transplantation (HSCT), and metabolic inhibitors are the examples of different therapeutic regimens combined with CAR-T cell therapy that has been discussed in detail in the following sections (Table 1). These combinatorial treatment approaches enhance the CAR-T cell safety and efficacy by modifying the tumor microenvironment, optimizing the CAR structure, bridging the CAR-T cells to the tumor cells, possibly targeting the multiple antigens, bypassing the tumor-immune escape mechanisms, and limiting the toxicity of CAR-T cell therapy (Fig. 1).

**Table 1:** Summary of CAR-T cell combination therapy clinical trials with other cancer treatments

Treatment	Combination strategy	CAR T cell target Ag	Phase	References
Immunomdulators	Decitabine	CD19/CD20	I, II	NCT04697940, NCT04553393
		CD19/PD-1	I	NCT04850560
	Lenalidomide	BCMA	I	NCT03070327
BiTEs	Obinutuzumab, glofitamab	CD19	II	NCT04703686
	Obinutuzumab, glofitamab, mosunetuzumab	CD19	II	NCT04889716
PD-1 inhibitor	PD-1 mAb	MUC1	I, II	NCT03525782
	Nivolumab, Pembrolizumab	CD30	I	NCT04134325
	Pembrolizumab	EGFRvIII	I	NCT03726515
		CD19	I, II	NCT02650999
	Nivolum	B cell Ag	II	NCT04205409
	Durvalumab	CD19	I	NCT02706405
	Tislelizumab	CD19/22	II	NCT04539444
PD-1 inhibitor CTLA4 inhibitor	Nivolumab, Ipilimumab	IL13Ra2	I	NCT04003649
Vaccine	Imovax Rabies	B cell Ag	I	NCT04410900
	VZV	GD2	I	NCT01953900
	PCV13	CD19	II	NCT04745559
Radiation	Apamistamab	CD19	I	NCT04512716
	Radiotherapy	B cell Ag	I, II	NCT04790747
		B cell Ag	NA	NCT04473937
Oncolytic virus (OV)	Binary OAV	HER2	I	NCT03740256
DC	peptide specific DC	B cell Ag	I	NCT03291444
rIL-1ra	Anakinra	CD19	II	NCT04359784, NCT04150913
BTK inhibitor	ibrutinib	CD19	I	NCT03960840, NCT02640209
	Acalabrutinib	CD19	I, II	NCT04257578
			II	NCT04484012
PI3-Kinase inhibitor	Duvelisib	B cell Ag	I	NCT05044039
Dimerizer drug	Rimiducid	P-PSMA	I	NCT04249947
Cytokine	IFN-α	B cell Ag	II	NCT04534634
	Aldesleukin (IL-2)	CD19	I, II	NCT00924326
		CD19/CD22	I, II	NCT03098355
Stem cell transplantation	HSCT	CD19	I	NCT03110640, NCT03685786
			II	NCT02794246
		CD19/CD20	II	NCT02846584
		CD123	I	NCT03114670
Chemotherapy Cytokine	Cyclophosphamide, fludarabine, Aldesleukin (IL-2)	IL13Rα2	I	NCT04119024
		EGFRvIII	I, II	NCT01454596
Chemotherapy CRISPR/Cas	Cyclophosphamide, fludarabine, CRISPR/Cas9	XYF19	I	NCT04037566
		CB-010	I	NCT04637763
Chemotherapy mAb	Cyclophosphamide, fludarabine, ALLO-647 (anti-CD52)	CD19	I	NCT03939026
			I, II	NCT04416984
		BCMA	I	NCT04706936
			I, II	NCT05000450
Chemotherapy mAb Immunomdulators	Cyclophosphamide, fludarabine, ALLO-647 (anti-CD52) Nirogacestat	BCMA	I	NCT04093596
Proteasome inhibitor Immunomodulator Corticosteroid mAb	Bortezomib Lenalidomide Dexamethasone Daratumumab	BCMA	II	NCT04133636
Immunomodulator Corticosteroid Antibiotic	Lenalidomide Dexamethasone clarithromycin	BCMA	III	NCT04287660
Chemotherapy Dimerizer drug	Bendamustine Fludarabine Cyclophosphamide AP1903	iC9-CD19	I	NCT03696784
Proteasome inhibitor Immunomodulator Corticosteroid Chemotherapy	Bortezomib Lenalidomide Dexamethasone Fludarabine Cyclophosphamide	BCMA	III	NCT04923893
Stem cell mAb Chemotherapy	HSCT Rituximab Fludarabine Cyclophosphamide	CD19	I, II	NCT01318317
Stem cell Chemotherapy	HSCT Carmustine, Etoposide, Cytarabine, Melphalan	CD19	I	NCT01840566
BTK inhibitor LAG3 inhibitor PD-1 inhibitor	Ibrutinib Relatlimab Nivolumab Durvalumab	B cell Ag	I, II	NCT03310619

*BiTEs* bispecific T cell engagers, *PD-1* programmed cell death protein 1, *CTLA4* Cytotoxic T-Lymphocyte Associated Protein 4, *DC* dendritic cell, *rIL-1Ra* recombinant IL-1 receptor antagonist, *BTK* Bruton's tyrosine kinase, *PI3* Phosphoinositide 3, *mAb* monoclonal antibody, *LAG3* Lymphocyte-activation gene 3, *BCMA* B cell maturation antigen, *MUC1* Mucin 1, *EGFRvIII* epidermal growth factor receptor variant III, *Her2* human epidermal growth factor receptor 2, *iC9* inducible caspase 9

### CAR-T cell in combination with chemotherapy

Chemotherapy can amplify the efficacy of CAR‑T cell therapy. Clinical research has shown that monotherapy is less efficient than a combination of chemotherapy and CAR-T cell therapy (Table 2). Each treatment alone cannot eradicate tumor cells and inhibit metastasis. Chemotherapy increases immune function while reducing tumor load. This method enhances the number of mature and active dendritic cells and elevates the migration of DCs and T cells into the tumor microenvironment. The chemotherapeutic agents induce the release of damage-associated molecular patterns (DAMPs) like ATP and high‑mobility group box 1 (HMGB1) by dying tumor cells [14]. Toll‑like receptor 4 (TLR4) is essential in recognizing DAMPs, which escalates DC maturation and activation [15, 16]. DC maturation can be induced by endogenous and exogenous type I interferon (IFN- I) as well, which are produced by tumor cells that have been exposed to chemotherapy [17].

**Table 2:** Summary of CAR-T cell combination therapy clinical trials with chemotherapy

Chemotherapy Drug	CAR-T cell Target Ag	Phase	References
Cyclophosphamide, fludarabine	CD19	I	NCT04653493, NCT03939026, NCT04037566, NCT04545762, NCT04892277, NCT03434769, NCT04732845, NCT02443831, NCT02529813
		I, II	NCT04404660, NCT03919240, NCT04416984, NCT03263208, NCT02685670, NCT00924326
		II, III	NCT03937544
	CD22	I	NCT04340167, NCT04088890, NCT04088864
	CD19/CD20	I	NCT03881761
	CD19/CD22	I	NCT04134325, NCT03919526, NCT04303520
		I, II	NCT04715217, NCT03287817, NCT03289455, NCT05225831, NCT04029038, NCT03448393
	CD19/BCMA	I	NCT03706547
	CD19/CD34	I	NCT04214886
	CD7	I	NCT04572308, NCT04860817
	CD30	I	NCT02917083, NCT03049449
		II	NCT04083495
	CD33	I	NCT03126864
	TriPRIL	I	NCT05020444
	GFRα4	I	NCT04877613
	MESO	I, II	NCT03916679, NCT03799913
	CD44v6	I, II	NCT04097301
	APRIL	I, II	NCT03287804
	BCMA	I	NCT04637269, NCT04626752, NCT04706936
		I, II	NCT03322735
	IM73	I	NCT04766840
	TAG72	I	NCT05225363
	CS1	I	NCT03710421
	IL13Rα2	I	NCT04510051
	15.21.GPC3	I	NCT04715191
	CD138	I	NCT03672318
	GPC3	I	NCT02876978, NCT05003895
	B7-H3	I	NCT04897321, NCT04670068
		I, II	NCT05323201
	EGFRvIII	I	NCT02844062
		I, II	NCT01454596
	IM9	I, II	NCT05155215, NCT03173417
	PSCA	I	NCT03873805
	GPC3	I	NCT05003895
	MUC1	I	NCT04025216
	LCAR-C18S	I	NCT04467853
	LCAR-M23	I	NCT04562298
	GD2	I	NCT04099797, NCT04196413, NCT02761915, NCT03721068, NCT03635632
		I, II	NCT03373097
	Senl-h19	NA	NCT04792593
Cyclophosphamide, fludarabine, AP1903	iC9-CD19	-	NCT03594162
Fludarabine, Melphalan, Cytoxan	CD5	I	NCT03081910
	CD7	I	NCT04264078
	CD19	I	NCT04264039
Gemcitabine, Capecitabine	CEA	II, III	NCT04037241
Cytoxan, Fludara, Keytruda	iC9-GD2	I	NCT01822652
FOLFOX, FOLFIRI	NKG2D	I	NCT03692429
Temozolomide	B7-H3	I, II	NCT04077866, NCT04385173

*BCMA* B cell maturation antigen, *GFRα4* glial-derived neurotrophic factor (GDNF) family receptor alpha 4, *MESO* Mesothelin, *APIRL* a proliferation-inducing ligand, *TAG72* Tumor-associated glycoprotein 72, *GPC3* Glypian-3, *PSCA* Prostate stem cell antigen, *MUC1* Mucin 1, *EGFRvIII* epidermal growth factor receptor variant III, *iC9* inducible caspase 9, *CEA* Carcinoembryonic antigen, *NKG2D* natural killer group 2, member D

In addition, chemotherapy reduces the activity of immunosuppressive cells like regulatory T cells (Tregs) and myeloid‑derived suppressor cells (MDSCs) as well as immunosuppressive agents like IL-10 and Transforming growth factor beta (TGF-β) cytokines. Maher et al. showed that chemotherapy with a standard carboplatin dosage had a synergistic effect on CAR-T cells that target Erb-B and sensitized tumor cells to degradation [18]. According to some research, chemotherapeutic agents have more effect on immunosuppressive cells than normal T‑cells, which are not harmful to T-cells [19]. Using chemotherapy before other therapeutic approaches can intensify the immunogenicity of T cells and increase the likelihood of lymphocyte existence in TME [20]. According to some investigations by Brentjen’s and Curran's groups, primary chemotherapy with cyclophosphamide (CTX) drastically increases the persistence and response of CAR19-T cell [21]. Except for this, chemotherapy can sensitize tumor cells against immunotherapy. For example, it elevates the amount of mannose‑6‑phosphate (M6P) receptors on tumor cells, which subsequently leads to more penetration of cytotoxic T cells (CTLs) into the tumor site by releasing granzyme B following the autophagy [22, 23].

Co-administration of chemotherapy with CAR-T cells inhibits the autoimmunity and immunosuppressive cells to enhance the persistence of CAR-T cells in vivo. This leads to a proper balance and maximizes the efficacy of both treatments [20, 24]. So far, the effect of chemotherapy on CAR-T cell therapy has been discussed more. Besides, a study demonstrated that CAR‑T cells could augment the efficacy of chemotherapy too. As Wang et al. noted, adaptive T-cells can reduce and even abolish the resistance of ovarian tumor cells to chemotherapy by releasing IFN‑γ. They observed that glutathione and cysteine, made by fibroblasts, contribute to this chemoresistance, and IFN‑γ can change the metabolism of fibroblasts through the Janus kinase 1/signal transduce and transcription 1 signaling pathway activator (JAK/STAT). Therefore, CAR-T cells can abrogate chemoresistance [25]. Chemotherapeutic drugs like cyclophosphamide, doxorubicin, fluorouracil, and others have been found to enhance the efficacy of CAR-T cell therapy through a variety of methods [9, 26]. In addition to increasing the synthesis of chemokines at the tumor site and the penetration of these CAR-T cells into cancerous cells, chemotherapy also makes the tumor cells more sensitive to granzyme B, allowing it to infiltrate the layer of tumor cells more readily. Chemotherapy increases the delivery of antigens. Additionally, it makes it simpler for CAR-T cells to recognize them [14].

Cancer-related macrophages are stimulated by immunogenic chemotherapy to create chemokines that help CAR-T cells adhere to lung tumors. A recent study showed that continuous CCL5 synthesis by human cancerous cells attracted the first wave of T cells that released IFN-γ upon antigen identification. This action triggered macrophages and DCs to secrete CXCL9, which attracted the second wave of CXCR3 + T cells [27, 28]. Chemotherapy also improves the toll-like receptor's ability to deliver tumor antigens to T cells by stimulating that receptor's activity. According to current research, several chemotherapy drugs, such as taxanes and vinca alkaloids, can make it easier to identify tumor cells by increasing calreticulin exposure and destroying tumor cells by creating a lot of tumor antigens [29].

In order to boost the effectiveness of humanized anti-CD19-CAR-T cells, individuals with refractory/relapsed (R/R) acute lymphoblastic leukemia are given extensive lymphodepleting chemotherapy regimens before receiving anti-CD19-CAR-T cell therapy (B-ALL). Cyclophosphamide alone, fludarabine added to cyclophosphamide, and bendamustine-based regimens are all parts of lymphodepleting chemotherapy. It has been demonstrated that lymphodepleting chemotherapy, which primarily aims to lower host T cells, also prolongs R/R B-ALL patients' event-free survival (EFS) time by promoting the proliferation and stability of anti-CD19-CAR-T cells [30, 31]. This information sheds new light on intensifying the potential of CAR‑T cells in the treatment of solid tumors and opens new perspectives for further research into the functional significance of these methods.

### CAR-T cell in combination with radiotherapy

Radiotherapy has been used as an excellent adjunctive treatment along with some monotherapies like checkpoint inhibitors and tumor vaccines [32]. Also, it can provide a tumor microenvironment that promotes CAR T-cell infiltration and trafficking into tumor sites [33]. So far, combination therapies, especially radiotherapy, have tamed the tumor microenvironment and overcome many TME barriers [34].

Radiotherapy contributes to the rise of MHC class I expression, which increases peptide synthesis and antigen presentation to maximize recognition by cytotoxic T cells and escalate the efficacy of adoptive CTL immunotherapy both locally and distantly [35]. According to evidence, radiotherapy can make tumor cells more sensitive to tumor-specific cytotoxic lymphocytes, making them more accessible to tumor-specific cytotoxic lymphocytes. This will lead to eradicating the tumor cells [35, 36]. Radiation not only stimulates the release of pro-inflammatory cytokines like IL-6, IL-1α/β, IFN-α/β, and Tumour necrosis factor α (TNFα) but also it helps to increase the release of IFN-γ and DAMPs, which induce the entrance of immune effector cells to the TME enhancing their function [37]. In addition to these, local radiotherapy may trigger the expression of particular chemokines, such as (CXCL) 1, 2, 9, 10, and 16, to boost the entry of T cells into the TME [38].

What makes radiotherapy unique is its dual feature against tumors. Not only do radiations cause immunity against tumors by stimulating CTLs on the local side but also, they can provide an inhibitory effect against distant tumors. Therefore, in addition to the regional anti-tumor development, metastasis suppression can be performed remotely [39]. CAR-T cell therapy can boost T-cell responses; thus, the anti-tumor outcomes should be augmented by pairing radiotherapy with CAR-T cell therapy [40]. There are several clinical trials of CAR-T cells combined with other treatments. For instance, a study at Duke University (NCT02664363) attempted to check out the safety and efficacy of epidermal growth factor receptor variant III (EGFRvIII) CAR‑T cells paired with radiation therapy. As reported by the authors, radiotherapy has additive or synergistic effects on CAR-T cell therapy and can be a promising treatment for solid tumors. Preliminary results of an investigation by Weiss et al. indicate that radiotherapy can enhance the function of CAR T cells in solid tumors. According to this study, radiotherapy intensified synergistically NKG2D expressing CAR-T cells (Natural killer group 2D receptor) in an orthotopic mouse model of glioblastoma. NKG2D binds to multiple ligands, which on the one hand, may decrease the likelihood of antigen escape, but on the other hand, it way exacerbates off-tumor killing by binding to non-malignant cells [40].

Based on a study by De Selm et al., antigen escape can lead to failure to treat solid tumors with the monotherapy method. They studied an orthotopic pancreatic cancer type that contained 25% cells that were devoid of sialyl Lewis A, the tumor-associated antigen for the CAR T cells used throughout the experiment. Better responses and higher rates of treatment were seen to combined therapy of low-dose sensitizing radiation and CAR-T cell therapy that was due to proapoptotic TNF-related apoptosis-inducing ligand (TRAIL) produced by activated CAR-T cells, while results of CAR-T cell monotherapy were not satisfactory. This phenomenon reinforces the theory that the combination of radiotherapy and CAR T cells can overcome antigen escape and dramatically eradicate tumor cells by increasing the expression of antigens [41]. As long as the presentation of antigens by dendritic cells is maintained, the tumoricidal responses of T lymphocytes persist. The mechanisms of the immune system are improved by radiation; it can be sensibly presumed that radiotherapy may have enhancing and even synergistic effect on the treatment regimen, which suggests that there is a need for continued research on the topic [41, 42].

### CAR-T cell in combination with oncolytic virus

Oncolytic virotherapy (OVT) is an emerging cancer immunotherapy approach that uses cancer killer viruses to infect tumor cells selectively. Because of prominent features of oncolytic viruses (OVs), like; capability of genetic modifications, selectively and directly cell targeting, and recruiting of the innate and adaptive immune system to induce an endogenous tumor-specific immune response [43], they can be employed to improve anti-cancer therapy in various aspects; (1) they can present tumor-associated antigens (TAA) to the immune system as a vaccine, (2) they can be genetically engineered to deliver exogenous therapeutic genes to be expressed in intratumoral milieu (3) they can also be combined with other immunotherapies like CAR-T cell therapy [44]. Altogether, OVT has the potential to synergize the efficacy of CAR T cells in solid tumors by spreading TAA upon the direct lytic effect on tumor cells which prevents antigen loss, reversing the tumor-mediated immunosuppression, which improves CAR-T cell persistence in the tumor microenvironment, and arming with potent therapeutic molecules like chemokines [45]. Therefore, different options can be installed on OVs to circumvent the CAR-T cell therapy barriers in solid tumors. Several pre-clinical studies have combined armed OVs with CAR-T cell therapy and have investigated their biological mechanism and effects (Table 3).

**Table 3:** CAR-T cell combination therapy with oncolytic viruses

Approach	OV name	CAR T-cell target Ag	Method	Outcome	References
Different modifications of Ovs which have been combined with CAR T-cell therapy					
Encode chemokines	Onc.Ad-IL15/RANTES	GD2	Intratumorally administration of OVs expressing the chemokine RANTES and the cytokine IL15, in neuroblastoma xenograft mouse model	Improving the migration and persistence of CAR T cells and enhancing the overall anti-tumor activity of CAR T cells	[40]
	VV.CXCL11	Meso	Intravenous injection of oncolytic VV expressing the chemokine CXCL11, in murine lung cancer model	Increasing trafficking of CAR T cells into tumors and enhancing anti-tumor efficacy of CAR T cell therapy	[41]
Encode cytokines	OAd-TNFα-IL2	Meso	Intratumoral injection of OV expressing the cytokines IL-15, IL-2, and TNF, in human-PDA-xenograft immunodeficient mice	Induced significant tumor regression and enhance the efficacy of CAR T cell therapy	[42]
	rAd.sT	Meso	Intratumoral injection of OV expressing TGF-β-antagonizing molecules, in triple-negative Breast cancer xenograft mouse model	Produced much more impressive antitumor responses to cancer and its metastasis (long-term anti-tumor response)	[43]
Engineered with immune checkpoint blocking agents	CAd-VECPDL1	HER2	Intratumoral injection of OV expressing a PD-L1 blocking mini-antibody, in HER2(+) prostate cancer xenograft models	Improved anti-tumor activity and controlled tumor growth	[45]
	CAd12_PDL1	HER2	Intratumoral injection of OV expressing a PD-L1 blocking antibody and IL-12p70, in head and neck squamous cell carcinoma xenograft models	Augmented the anti-tumor effects of adoptively transferred CAR T cells and controls the growth of both bulky and metastasized tumors	[46]
Delivery of tumor-selective surface antigens	OV19t	CD19	Intratumoral (local) and intraperitoneal (reginal) injection of oncolytic VV expressing de novo CD19 at the cell surface, in several mouse tumor models	Promoted tumor control	[49]
Expression of bispecific T-cell engagers (BiTE)	OAd-BiTE	FR-α	Intratumoral injection of OV expressing BiTEs specific for EGFR, in mouse xenograft models of cancer	Improved antitumor efficacy and prolonged survival	[50]
	CAdTrio	HER2	Intratumoral injection of OV expressing a PD-L1 blocking antibody, IL-12p70, and BiTEs specific for CD44, In several xenograft models	Improved potency, duration, and breadth of antitumor activity of CAR T cells	[51]
	EphA2-TEA- VV	HER2	Injection of oncolytic VV expressing secretory bispecific antibodies that bind both to CD3 and a tumor cell surface antigen EphA2, In an orthotopic lung tumor xenograft model	Reduced significantly tumor growth	[52]
Laoding OV into cell	VSVΔM51	HER2	Loaded chimeric antigen receptor–engineered T cells with OV in vitro	Successfully deposited virus onto tumor targets and this combination has the potential to enhance the efficacy of each of the two approaches	(53)

*OV* Oncolytic virus, *Onc.Ad* Oncolytic adenovirus, *OAd* Oncolytic adenovirus, *RANTES* regulated on Activation, Normal T Cell Expressed and Secreted, *VV* vaccinia virus, *BCMA* B cell maturation antigen, *GFRα4* glial-derived neurotrophic factor (GDNF) family receptor alpha 4, *MESO* Mesothelin, *PD-L1* programmed cell death protein ligand 1, *EphA2* Erythropoietin-producing Hepatocellular receptor tyrosine kinase class A2, *BiTE* bispecific T cell engager, *Her2* human epidermal growth factor receptor 2, *TNF-α* Tumor necrosis factor α

One of the major obstacles to CAR-T cells is limited migration into the tumor site [7]. Nishio et al. investigated the therapeutic effect of GD2-CAR-T cells in combination with oncolytic adenovirus (OAds) expressing the RANTES and IL-15 in the neuroblastoma xenograft mouse model. Results showed a synergistic effect of OVT on CAR-T cell therapy. Accordingly, RANTES and IL-15 increased the accumulation of CAR T-cells at tumor sites and enhanced the sensitiveness of tumor cells to the lytic effects of CAR-T cells [46]. Similar results were accrued when CAR-T cells combined with oncolytic vaccinia virus (VV) expressing CXCL11 (a potent chemokine that attracts T cells to the tumor sites). It was also shown that CXCL11 delivering to the tumor site by a VV is more efficient in tumor cell killing in vivo than secreting CXCL11 by CAR-T cells [47]. Another study demonstrated that the combination of CAR-T cells and modified OVs expressing TNF-α and IL-2 has a robust anti-tumor effect in the pancreatic ductal adenocarcinoma (PDA) murine model [48]. Regardless of the strong immunosuppressive TME of PDA and its prone to metastasizing, the combination therapy could successfully modulate the TME and prevent metastasis. That was exerted by promoting the M1 polarization of macrophages and the maturation of DCs. In addition to the increased production of pro-inflammatory cytokines via OV, the inhibition of anti-inflammatory cytokines and their effects on CAR T-cell therapy were evaluated by Y. Li et al. They examined the OAds expressing soluble receptor of TGF-β (an important immunomodulatory factor that inhibits anti-tumor immunity in TME) (rAd.sT) in a triple-negative Breast cancer xenograft mouse model. They found enhanced anti-tumor immune responses combined with CAR-T cells [49].

Prescription of immune checkpoint inhibitors (ICIs) is another beneficial cancer treatment regimen, but systemic administration of ICIs causes immune-related side effects. On the other hand, local administration of ICIs leads to the limited distribution of metastasized tumors [50]. To solve these challenges, Tanoue et al. engineered an oncolytic adenovirus (Onc.Ad) with a helper-dependent adenovirus (HDAd) expressing a Programmed death-ligand 1 (PD-L1) blocking mini-antibody (CAd-VECPDL1). They evaluated its anti-tumor efficacy in combination with human epidermal growth factor receptor 2 (HER2)-specific CAR-T cells. They demonstrated that intratumoral administration of CAd-VECPDL1 combined with CAR-T cell therapy augments the anti-tumor effect of HER2-CAR-T cells and significantly prolonged animal survival in a prostate cancer xenograft mouse model compared to separated monotherapies [51].

Furthermore, Rosewell Shaw et al. added another option to the previously mentioned strategy by engineering an OV that could secrete IL-12p70 in addition to the PD-L1-blocking antibody to promote the anti-tumor efficacy of CAR-T cell therapy for head and neck cancer. In other words, they evaluated a process that could simultaneously provide oncolysis, checkpoint inhibition, and pro-inflammatory cytokine production. The mentioned strategy could effectively control the growth of both bulky and metastasized tumors, whereas Ad virotherapy or ICI alone could not handle it [52]. For efficient tumor cell eradication, stimulation of CAR-T cells by cytokines and chemokines is not sufficient and selective engagement of CAR-T cells with tumor cells is necessary [53]. On the other hand, tumor antigen heterogeneity, tumor antigen loss, and TAA presence in some normal tissues have limited the effective anti-tumor responses of CAR-T cells [54]. In this regard, Park et al. developed an oncolytic VV (OV19t) expressing a truncated non-signaling variant of CD19 (CD19t) on multiple tumor types to enhance CD19-specific CAR-T cells activity. It was found that tumor cell infection following regional administration (intraperitoneal injection) of OV19t led to impressive CAR-T cell engagement through CD19t. CD19t was selectively expressed on tumor cells, unlike non-tumoral cells, and induced tumor-specific immune memory by embroiling endogenous anti-tumor immunity [55].

### CAR-T cell in combination with cancer vaccines

Cancer vaccines are immunotherapeutic approaches that can optimize immune response by disposing of tumor-associated epitopes and inducing an adaptive immune system as a target tool. So, combining cancer vaccines with CAR-T cells can be a proper solution to overcome some limitations of CAR-T cell therapy like downregulation of target antigen, exhaustion, etc. [60]. Vaccines can promote CAR-T cells through two main approaches. The first strategy is to stimulate CAR-T cells via Antigen-presenting cells (APCs) or a human leukocyte antigen (HLA)–dependent manner. The second strategy is direct stimulation of dual or bi-specific CAR T-cells into the tumor site [61]. According to the structure of vaccines, they are divided into cellular, molecular, and virus-based vaccines [12] (Table 4).

**Table 4:** CAR-T cell combination therapy with cancer vaccines

Vaccine Classification		Vaccine	CAR T-cell ligands		Outcome	References
Type	Approach		CAR ligand	TCR ligand		
Cellular	Engineered cell	K562-derived whole-cell	GD2	CMV	safely enhanced antitumor effect of adoptively transferred CAR-redirect CMV-CTLs	[63]
		LCL	CD19	EBV	enhanced the persistence and expansion of CD19CAR CTL	[64]
	DC stimulation	WT1 peptide vaccine	WT1	_	Available targets to CAR-T cells can be expanded and efficacy of CAR-T cells boosted by vaccination	[66]
		Eps8-DCs	CD19	–	﻿Eps8-DCs exerted significantly synergistic effect on CD19 targeting CAR-T cells	[75]
Molecular	Tailored nanoemulsion	OVA-Clec9A-TNE	Her2	OVA	Improved CAR T-cell proliferation and inflammatory cytokine secretion in vitro and durable responses and some rejections of tumors in vivo	[67]
	Amph-ligand vaccine	Amph-FITC	FITC	–	Boosted triggered massive CAR-T cells expansion, increased donor cell polyfunctionality, and enhanced antitumor efficacy	[76]
	Nanoparticulate RNA vaccine	CLDN-LPX	CLDN 6	CLDN 6	Improved engraftment of CAR-T cells and regression of large tumors	[68]
Viral	Viral peptide	CMV peptide	CD19	CMV	The bispecific T cells exhibited proliferative response and enhanced antitumor activity following CMVpp65 peptide vaccine administration	[73]
	Engineered virus	VV-gp100	Her2	gp100	Induced expansion of CAR T cells and their localization to tumors, which led to eradication of large established solid tumors of several histologies in mice	[77]

*VV* vaccinia virus, *AMPH* amphiphile, *LCL* Lymphoblastoid cell lines, *WT1* Wilms’ Tumor Gene 1, *Eps8* Epidermal growth factor receptor pathway substrate 8, *DC* dendritic cell, *CLEC9A* C-type lectin domain family 9A, *TNE* tailored nanoemulsion, *OVA* ovalbumin, *FITC* Fluorescein isothiocyanate claudin, *CLDN* *CMV* Cytomegalovirus, *Her2* human epidermal growth factor receptor 2, *EBV* Epstein-Barr virus, *gp100* Glycoprotein 100

#### Cellular vaccines

In the cellular vaccines platform, the whole cell or cellular components are applied as an antigen source and a carrier of antigen to APCs. Different cells can be exploited in cellular vaccines, including tumor cells, irradiated immortalized cell lines, and dendritic cells (DCs) [62]. The first study which tried to evaluate the combination of CAR-T cells with a whole-cell vaccine was performed by Caruana et al. They developed the K562 tumor cell line expressing a highly immunogenic protein, CMV-pp65. This cellular approach benefits from the faster cross-presentation of viral epitopes on APCs and can easily be manipulated ex vivo to express costimulatory molecules like CD40L and OX40L. In addition, they engineered a dual CAR-redirected virus-specific cytotoxic T cell (CAR-CMV-CTLs) by transduction of GD2-specific CAR into cytomegalovirus (CMV)-cytotoxic T cells (CTLs). The vaccine led to better in vivo anti-tumor effects of adoptively transferred CAR-CMV-CTLs among xenograft models of neuroblastoma without any significant side effects [63]. In phase I/II multi-center trial of CD19-CAR-CTL therapy in children with ALL conducted by Rossig et al., the Donor-Epstein-Barr-virus-specific cytotoxic T-cells (EBV-CTL) were transduced to the CD19-CAR. The irradiated EBV transformed lymphoblastoid cell line (LCL) was utilized as a cellular vaccine. Results revealed that vaccination with donor EBV LCL could enhance the persistence and expansion of CD19-CAR-CTL [64].

DCs are the connector and regulator of innate and adaptive immune responses and have important roles in the anti-tumor immune response by processing and presenting tumor antigens to T cells [65]. To induce immunological memory and prevent tumor relapse, a DC-based vaccine has been developed through ex vivo activation of DCs by loading specific antigens [62]. Since tumor relapse is one of the obstacles to CAR-T cell therapy in hematological malignancies, combining CAR-T cells with DC-based vaccines would increase the anti-tumor function. In this case, Wu et al. generated the Eps8-DCs by pulsing DCs with HLA-A2.1-specific Epidermal growth factor receptor (EGFR) pathway substrate 8 (Eps8)-derived peptides and then co-cultured with CD19-CAR-T cells. It was found that the vaccine led to longer in vitro persistence, better expansion of CAR-T cells, synergistic effect on cytolysis of Eps8 + tumor cells, and strong capacity to produce IL-2 (a substantial marker of CAR-T cell potency and persistence) and TNF-α (a vital cytokine for the induction of anti-tumor cellular immunity) upon stimulation with CD19 + leukemia cells [66].

#### Molecular vaccines

Molecular vaccines have been under investigation because of the difficulty and expensiveness of cellular-based vaccine production. Different molecules are involved in vaccine therapy against cancers, such as peptides, RNA, and DNA. Most of them follow the same mechanism for loading specific antigens into APCs and inducing T cells. Therefore, CAR-T cell efficacy can be boosted by developing the sensitive CAR-T cell to target tumor cells by MHC-directed antigen through the vaccine. The critical issue of this approach is the proper delivery of the vaccine to APCs in the secondary lymphoid organs. So, designing and utilizing an efficient vehicle is more important. Accordingly, Chan et al. combined a nanoparticulate vaccination platform with CAR-T cell therapy. They encapsulated ovalbumin (OVA) peptides into Clec9A-tailored nanoemulsion (OVA-Clec9A-TNE) to deliver OVA to APCs. Then, dual-specific CAR, so-called CAROT, was stimulated by either cross-presented MHC-directed OVA via own OVA-specific TCR or HER2 positive tumor cells via own HER2-CAR. The adoptive transfer of CAROT cells in combination with the OVA-Clec9A-TNE vaccine was found to durable remission of solid tumors in mice. It was exerted by significant proliferation and anti-tumor activity of CAROT cells, high levels of tumor-infiltrating DCs, and permanent immune response memory [67].

In another investigation, Reinhard et al. recruited the combination of nanoparticulate vaccine with CAR-T cell therapy. They generated liposomal antigen-encoding RNA (RNA-LPX) by mixing anionic mRNA with cationic liposomes [68]. This RNA act as either adjuvant due to its counteraction with Toll-like receptor (TLR), which leads to upregulation of costimulatory molecules, or antigen due to augmentation of antigen expression on the surface of DCs [69]. According to this goal, they used claudin 6 (CLDN6) as the same target antigen for CAR-T cells and expression on DC's surface following the vaccination. As the result of the combination component, so-called CARVac, superior expansion and cytolysis activity of CAR T-cells were found in vivo, which led to better control of tumors [68]. The most noticeable point of this study was presenting such a feasible, applicable, and economic strategy for vaccine production to attenuate CAR-T cell therapy obstacles in solid tumors.

#### Viral vaccines

Considering the feasibility of an agent to boost CAR-T cell therapy, viral based vaccines are the best choices. using the complete viruses or just their antigen as immunogen to promote virus-sensitive T-cells has been investigated in various studies [70–72]. Wang et al. transduced CD19 CAR into the T cells upon stimulation by CMVpp65 protein to produce CD19-CAR-redirected CMV-specific T cells. as discussed before, these bispecific T cells could be activated by CD19 via CAR engagement as well as CMV peptides antigens via TCR engagement in a HLA-dependent manner [73]. they reported that because both TCR and CAR located on same T cells, vaccination with CMVpp65 enhanced anti-tumor activity of CAR T-cells and illustrated that this approach could be expanded to a wide range of malignancies in patients who don’t respond CAR-T cell therapy alone. in a similar strategy, Slaney et al. applied a manipulated Recombinant vaccinia viruses encoding human gp100 (VV-gp100) in combination with Her2 CAR-T cells [74]. in contrast to CMV peptide vaccine, VV-gp100 lysed tumor cells and induced inflammation which indicate VV-gp100 could regress tumor through several ways. also, they accommodated dual-specific T cells, recombinant vaccinia, and high dose of IL-2 in the single strategy named Adoptive Cell Transfer Incorporating Vaccination (ACTIV) therapy. overall, ACTIV therapy led to extensively expansion of dual-specific T cells and partially resistant to rechallenging with different tumors in surviving mice after primary tumor rejection.

### CAR-T cell combination therapy with cytokines

Cytokines are pleiotropic signaling molecules involved in numerous immunologic processes. They are key modulators of immunologic responses, making them promising candidates for co-administration with CAR-T cells. Tumors can evade the immune system via producing immunosuppressive cytokines, such as IL-4. Hence, developing IL-4-resistant CAR-T cells might potentially enhance their efficacy. It was found that employing inverted cytokine receptors (ICRs) could inhibit the IL-4 immunosuppressive functions in IL-4 positive tumors. Recent investigations indicated that merged forms of ICRs potentially enhanced the efficacy of ICR-CAR-T cells. For instance, Mohammed et al. [78]. developed specific CAR-T cells against prostate stem cell antigen (PSCA) to target pancreatic cancer. In this case, they designed an ICR via incorporation of IL-4 receptor exodomain and IL-7 receptor endodomain (4/7 ICR). They reported that PSCA-CAR-T cells could effectively kill tumor cells; however, they were inadequately extended in the immunosuppressive milieu of the tumor. In addition, they observed that transgenic expression of ICR in T cells induced the proliferation of T cells in TME but no anti-tumor responses. In comparison, transgenic co-expression of 4/7 ICR and CAR in T cells (expression of 4/7 ICR and CAR expression in PSCA-CAR-T cells) increased T cell expansion and anti-tumor activities.

In line with attempts to enhance CAR-T cell potency against the immunosuppressive microenvironment of solid tumors, a novel 4/21 ICR was developed by fusion of IL-4 receptor ectodomain and IL-21 receptor endodomain. The 4/21 ICR structure was inspired by the 4/7 ICR design; however, the underlying action mechanisms differed from the 4/7 ICR. IL-4 engagement induced 4/21 ICR activation, leading to STAT3 phosphorylation [79]. Enhanced STAT3 phosphorylation serves as a remarkable index for CAR-T cell capacity. In patients with an appropriate response to CAR-T cell therapy, STAT3 genes were highly expressed. The activated STAT3 by 4/21 ICR increased the CAR-T cell resistance to immunosuppressive effects of IL-4 and mediated the anti-tumor function of CAR cells [80]. Interestingly, the cytotoxic activity of 4/21 ICR-CAR-T cells was preserved upon the IL-4 presence. Also, 4/21 ICR-CAR-T cells displayed a Th17-like phenotype in vitro. Thus, it is concluded that despite the immunosuppressive profile of IL-4, it could enhance CAR-T cell potency via developing a Th17-like phenotype [79].

IL-7 plays a crucial role in enhancing effector immune responses. IL-7 selectively promoted the growth and expansion of memory and naive T cells. Due to lacking IL-7Rα receptor, Treg cells are irresponsive to IL-7, so IL-7 cannot promote Treg cell expansion. For this reason, IL-7 could selectively promote anti-tumor responses of T cells, while immunosuppressive activities of Treg cells, if not decreased, remained intact. Thereby, preserving T cell responsiveness to IL-7 by engineering specific CAR-T cells has been considered. To this end, CAR-T cells expressing the IL-7Rα subunit (IL-7Rα-transgenic/CAR-redirected EBV-CTLs) were engineered and investigated against neuroblastoma cells in vivo and in vitro [81]. Findings revealed a selective immune augmentation effect of IL-7 on CAR-T cells, including enhanced anti-tumor function, expansion, and persistence. In contrast, present Treg cells were not affected. Thus, IL-7, as a good factor, could selectively augment immune responses of the redirected CAR-T cells and/or intended effector cells; meanwhile, overcome immunosuppressive features of Treg cells. Moreover, a study reported that AXL-CAR-T Cells exerted potent anti-tumor cytotoxicity against AXL-positive triple-negative breast cancer (TNBC) [82]. Similarly, another study showed that the co-expression of the IL-7 receptor (C7R) in AXL-CAR-T cells enhanced the expansion of CAR-T cells against TNBC. The C7R-AXL-CAR-T cells significantly promoted the cytotoxicity and survival of the CAR-T cells compared to the AXL-CAR-T cells (without IL-7 receptor). Thus, C7R expressing AXL-CAR-T cells could overcome the barriers of the AXL-CAR-T cells in solid tumor treatment [83].

IL-15 is another potent immunostimulatory cytokine against tumor cells. IL-15 exerts a crucial role in CD8 + T cell trafficking, T cell persistence, and improving memory T cell generation [84]. IL-15 gene and an inducible caspase-9-based suicide gene emerged to generate safe iC9/CAR.19/IL-15 + T cells against lymphoma and leukemia. The reports showed an enhanced CAR-T cell anti-tumor activity and proliferation as well as a significantly reduced tumor cell growth. Also, IL-15 downregulated the PD-1 receptor expression and prevented CAR-T cell exhaustion [85]. In a study performed by Alizadeh et al. [86], combined CAR-T cell with IL-15 was investigated against lymphoma and glioblastoma models. As a result, CAR-T/IL-15 cells decreased mTORC1 activity and subsequently preserved the stem cell memory T cell (TSCM) phenotype of CAR-T cells with lesser differentiation. Further, the coexpression of murine IL-15 (mIL-5) upregulated antiapoptotic molecule Bcl-2 and decreased PD-1 expression, which enhanced the function, survival, and proliferation of CAR-T cells and made them resistant to exhaustion and apoptosis [87].

A study in 2021 compared the effects of ex vivo cytokines in function and persistence of BCMA-CAR T cells in in vivo murine model of MM in three settings: IL-15 alone, IL-2 alone, and IL-15/IL-7 combination. Interestingly, the IL-15-cultered BCMA-CAR T cells showed limited differentiation and dysfunction, which lead to further improvement in survival and efficacy of CAR T cells compared to CAR T cells cultured with IL-2 or IL-15/IL-7 [88]. This suggests single IL-15 could be a better candidate than IL-2 or IL-15/IL-7 combination for MM CAR T cell therapy.

In addition, IL-15 improved CAR-T cell metabolic profile by promoting mitochondrial potential and glycolytic enzyme downregulation. Interestingly, CAR-T/IL-15 cells decreased 2B4 and Lymphocyte activation gene-3 (LAG3) expression, which serve as inhibitory molecules. TSCM phenotype was also preserved and emerged using second-generation CD19 mbIL-15-CAR T cells, which were engineered to express a membrane-bound chimeric IL-15 (mbIL-15). To prevent adverse effects and toxicities, mbIL-15 was tested instead of exogenous IL-15. mbIL15 signaling exerted durable persistence of the TSCM phenotype and enhanced CAR-T cell survival and activity. Moreover, IL-15 prevented CAR-T cell exhaustion via downregulation of the exhaustion makers, such as PD-1, LAG3, and 2B4. IL-15 also could enhance CAR T cell metabolism [89].

### CAR-T cell combination therapy with Checkpoint inhibition

The checkpoint signaling serves as a regulatory means of balancing immune responses via downregulating excessive immune activities, which impedes autoimmunity. Hence, checkpoint blockade (CPB) has been a growing field of interest in cancer immunotherapy. Checkpoint ligands derived from tumor cells mediate immune evasion through exhausting immune effector cells. CPB is a novel and efficient immunotherapy that employs agents known as immune checkpoint inhibitors (CPIs). The CPIs enhance immune system function and induce infiltration and persistence of immune cells. Various studies have reported the excellent outcomes of combination therapy involving CAR-T cell and PD-1 blockade. Therefore, combining CAR-T cell and PD-1 CPB has generated a growing field of interest in CAR-T cell combination therapy.

### Extrinsic CPB (PD-1/PD-L1 axis blocking antibodies)

It was found that the co-administration of pembrolizumab (an anti-PD-1 mAb) with CAR-T cell interestingly recovered TNF-α and IFN-γ production, while IL-2 production was not restored. The combination of anti-PD-1 CPB with CAR-T cell therapy enhanced CAR-T cell persistence and function in patients with metastatic melanoma [90]. In 2013, a study investigated the combination therapy of anti-Her-2 CD8 + T cells and anti-PD-1 antibody in murine breast cancer models. They reported that combining CAR-T cell therapy with an anti-PD-1 antibody could enhance tumor eradication and promote therapeutic outcomes. PD-1 blockade improved T cell immunity in vivo and in vitro via strengthening the activity of CAR-T cells and the expression of IFN-γ and granzyme B. Interestingly, there was no evidence for increased risk of autoimmunity in normal Her-2-expressing tissue [91]. Despite the outstanding outcomes of extrinsic anti-PD-L1 and CAR-T cell combination therapy, systemic administration of PD-1-blockade may cause multiple concerns. Anti-PD1 antibodies barely reach an optimum persistence and concentration level in TME when systemically infused. This will increase treatment costs while the optimum outcome is not achieved. In addition, as an immune-augmenting strategy, systemic PD-1-blockade may induce uncontrolled T cell activation. Also, several side effects have been reported that affect the endocrine system, pancreas, gastrointestinal tract, liver, and skin and cause renal failure, hypothyroidism, pancreatitis, hepatitis, colitis, and dermatitis [92, 93]. Hence, targeted CPB delivery via CAR-T cells appears to be a solution for unintended adverse effects. In addition, it has been shown that IL-7/IL-15-cultured CAR T cells elicited a better response to anti-PD-1 adjuvant therapy compared to IL-2/IL-7-culturd CAR T cells. Thus, combining anti-PD-1 CPB with CAR T cells cultured in a particular cytokine preconditioning milieu could enhance the therapeutic outcome [94]. This may reduce required anti-PD-1 dose, and decrease treatment-related adverse events.

#### Dominant-negative receptor (DNR) and short hairpin RNA (shRNA)

Both CD28 and 4-1BB CAR-T cells showed an appropriate increase in CAR-T cell persistence. After frequent antigen exposures, 4-1BB CAR-T cells appeared resistant and demonstrated sustained potency and cytotoxicity. 4-1BB CAR-T cells successfully eradicated the tumor at low dose administration. In contrast, CD28 CAR-T cells indicated significantly lower cytotoxicity than 4-1BB CAR-T cells, attributed to the PD-1 receptor overexpression by CD28 CAR-T cells. Cherkassky et al. [95]. designed and evaluated two second-generation mesothelin-specific CAR-T cells using CD28 and 4-1BB costimulatory signaling domain. They attempted to improve the CD28 CAR-T cell function by combining it with anti-PD-1 CPB. The results demonstrated that anti-PD-1 antibody enhanced CD28 CAR-T cell function and effectiveness. The optimum CD28 CAR T cells activity relied on frequent PD-1 antibody co-administration and might induce side effects. Thus, they genetically engineered CD28 CAR-T cells to express PD-1 dominant negative receptor (DNR), to overcome PD-1-mediated immunosuppression in a single administration. The PD-1 DNR contained an extracellular PD-L1 binding domain, lacked a signaling domain, and was functionally silent. When PD-1 DNR expression reached an optimum level, PD-1 DNR competed with endogenous PD-1 receptors and decreased PD-L1 engagement with PD-1. This process significantly neutralized PD-1-mediated immunosuppression and enhanced CD28 CAR-T cell function and persistence in vivo and in vitro.

In addition, they attempted to target and downregulate the PD-1 receptor at the protein level. For this purpose, they transduced CD28 CAR-T cells with PD-1–targeting shRNAs using vectors. It has been indicated that PD-1 shRNAs increased cytokine production, enhanced CAR-T cell proliferation, and invigorated anti-tumor cytotoxicity. It has been demonstrated that PD-1 DNR is further efficient than the PD-1 antibody or short hairpin RNA (shRNA) [95]. In a study of the shRNA mechanism, retroviral technology was utilized to target and knockdown adenosine 2A receptors (A2ARs) in anti-HER2 CAR-T cells. In this case, reducing the level of A2aR mRNA expression significantly enhanced CAR-T cell function [96]. Also, Cytotoxic T-Lymphocyte Associated Protein 4 (CTLA-4) was genetically knocked down using shRNA, which benefited CAR-T cell anti-tumor function. CTLA-4 knockdown enhanced IFN-γ and TNFα production and increased CAR-T cell expansion [97].

#### Gene edition

##### Single gene edition

Clustered regularly interspaced short palindromic repeats (CRISPR)/Cas9 is a novel technique used in editing cellular genome profiles. This technology could delete the gene loci involved in the expression of immune checkpoint receptors. In 2019, CAR-T cells were employed to target mesothelin, and the Programmed cell death protein 1 (PDCD1) gene locus was knocked out using the CRISPR/Cas9 technique. This process enhanced the CAR-T cell cytotoxicity and cytokine secretion against PD-L1 positive cells in vitro and inhibited the tumor relapse in vivo [98]. In a similar study, CRISPR /Cas9-mediated PDCD1 disruption increased the CD19-CAR-T cell cytotoxicity in vitro and improved CAR-T cell-mediated removal of tumor tissue in vivo in a xenograft tumor model [99]. Also, the knockout of LAG-3 was tested in lymphoma xenograft. Although the survival of the CAR-T cells was not significantly affected in vitro, CAR-T cell anti-tumor responses were augmented in vitro and in vivo [100].

##### Multiplex gene edition

Editing and altering multiple gnome profiles appears to be a potential strategy to overcome the limitations of generating universal CAR-T cells and enhancing the treatment's efficacy. Using allogeneic T cells to produce universal CAR-T cells instead of autologous T cells may overcome the insufficiency potency of autologous T cells in function and proliferation and save time and expense; however, graft-versus-host disease (GVHD) is a significant concern. Simultaneous CRISPR/Cas9-mediated knockdown of endogenous TCR and β-2 microglobulin (B2M: an essential subunit of the HLA-I) defeated the GVHD. In this context, multiplex gene edition has been a novel area of interest in CRISPR/Cas9-mediated CBP therapy. It has been shown that deletion of T Cell Receptor Alpha Constant (TRAC), B2M, and PDCD1 in universal EGFRvIII and CD19-CAR-T cells enhanced their anti-tumor responses, respectively, against both solid and hematologic malignancies (glioblastoma and leukemia). In addition, these triple-disrupted universal CAR-T cells did not elicit GVHD reactivity [101]. Four-gene-edited universal CD19-CAR-T cells were tested against Nalm6 leukemic cells (Nalm,6: A B cell precursor leukemia cell line). In addition to TRAC and B2M abrogation, Fas was also ablated to reduce Activation-induced cell death (AICD) of CAR-T cells and enhance their survival. This triple gene disruption generated apoptosis-resistant universal CAR-T cells. To strengthen CAR-T cells against checkpoint inhibition, double gene disruption of CTLA-4 and PD1 was accomplished. Results indicated the increased persistence and cytotoxicity as well as resistance to apoptosis and immune inhibition of CAR-T cells [102].

##### Costimulatory molecules

4-1BB and CD28 serve as costimulatory molecules in CAR-T cells. The effect of these costimulatory molecules has been tested and compared in previous studies. As reported previously, the combination of 4-1BB and CD28 costimulatory domains in CAR-T Cells showed differences in inhibitory molecule expression and CAR-T Cells exhaustion. It has been reported that CAR-T cell incorporation with 4-1BB, compared to CD28, indicated a significant reduction in the expression of inhibitory molecules, such as T cell immunoglobulin and mucin domain-containing protein 3 (TIM3), LAG-3, and PD-1 as well as the exhaustion-associated transcription factors. Thus, 4-1BB, compared with CD28, remarkably haltered CAR-T cell exhaustion and improved CAR-T cell function [95, 103].

##### Pharmacological antagonists

The pharmacological blockade was accomplished using the A2AR antagonist SCH58261. SCH58261 increased the expression of granzyme B in tumor-infiltrating CD8 + T cells. The combined treatment of SCH58261 and anti–PD-1 significantly promoted CAR-T cell function; however, the lonely administration of SCH58261 and anti-PD-1 antibody showed an insignificant increase in CAR-T cell function [96].

### CAR-T cell combination therapy with BiTEs

Generally, human antibodies are monospecific and target only one antigen, while bispecific antibodies like Bispecific T cell engager (BiTEs) can target different antigens. BiTEs recruit T cells into the tumor microenvironment to increase the efficacy in combating the tumor [104]. BiTE is a dual-specific antibody composed of two ScFvs from different antibodies. BiTEs can engage both T cell and tumor cells by binding to CD3 on CAR or bystander T cell (anti-CD3 mAb) and a target antigen on tumor cells (anti-TAA mAb), respectively. As an advantage, BiTE can link T cells and tumor cells without restriction to MHC and induce T cells' activation, proliferation, and cytotoxic function efficiently [105]. BiTEs connect CAR-T cells to different tumor cells, resulting in an efficient tumor-cell killing. Accordingly, Choi et al. designed and evaluated EGFRvIII specific CAR-T cell secreting EGFR-specific BiTE antibody (BiTE-CAR-T Cell) against glioblastoma mouse model, which indicated the potential efficacy in eliminating the heterogeneous tumor cells without any significant toxicity [106]. However, EGFRvIII specific CAR-T cell monotherapy could not eradicate the heterogeneous glioblastoma cells [53].

In one another encouraging published outcome, prescribing the blinatumomab, a BiTE, alongside anti-CD22 CAR-T cell led to the complete eradication of tumor cells and patients’ prolonged life span who relapsed after anti-CD22 CAR-T cell monotherapy [107]. CD3/EGFR BiTEs alongside an anti-EGFRVIII CAR-T cell was another example designed for neuroblastoma, which could successfully target the tumor cells expressing EGFR [106]. Shalabi et al. also investigated the efficacy of anti-CD19 CAR-T cells along with blinatumomab in a relapsed/refractory B-ALL case. In this case, loss of CD19 was the primary reason for relapse after receiving anti-CD19-CAR-T cell monotherapy. Interestingly, blinatumomab induced CAR-T cell expansion, proliferation, persistence, and cytokine production, which led to the potent defeat against tumor cells [106].

In another combination strategy, CAR-T cells can be accompanied by the simultaneous or sequential prescription of bispecific adapters consisting of conjugated antibody portions, known as tags (fluorescein isothiocyanate, biotin, etc.). Specific-antigen targeting is mediated by the collaboration of bispecific adapters (tags) against tumor antigens and anti-tag CAR-T cells. In this context, Lohmueller et al. designed an anti-biotin CAR-T cell and administered it with biotinylated bispecific antibody (anti-CD19 or anti-CD20) coated on tumor cells, which exhibited the higher induced function of CAR-T cell, IFN-γ production, and tumor cell eradication. The encoding sequences of BiTE and CAR can be transfected into T cells and lead to the production of armored BiTE-CAR-T cells with powerful features in targeting the heterogeneous tumor cells. Folate receptor (FR) is a tumor antigen expressed in several cancers, including lung, ovarian, uterus, and breast, with low expression levels on normal cells. Exploiting the folate- fluorescein isothiocyanate (FITC) conjugate, a bispecific small molecule switch, is another approach that controls the CAR-T cell function in the tumor site and can be used to target FR. In this way, an anti-FITC-CAR-T cell is composed of a binding region for a FITC molecule. The prescription of BiTE (folate-FITC conjugate) recruits the CAR-T cells and induces their function to target the tumor cells expressing the FR [108]. To redirect the anti-FITC-CAR-T cells to the target antigen on tumor cells, folate-FITC can conjugate to different anti-tumor antibodies (anti-CD19, anti-CD20, anti-CD22, anti-Her2, and anti-EGFR) and makes diverse Ab-FITC bispecific small molecules.

Consequently, this strategy keeps CAR-T cell function under control with higher efficacy and lower toxicity [109]. BiTEs and CAR-T cells would synergistically generate the strengthened ant-tumor responses as a promising combination treatment. Thereby, utilizing this kind of combinational therapy with CAR-T cells may overcome the loss of tumor antigen, antigen heterogeneity, and limited efficacy and persistence of CAR-T cells.

### CAR-T cell combination therapy with Immunomodulatory agents

Several supporting data recommend the combination treatment of CAR-T cells with immunomodulatory drugs, providing efficient tumor cell eradication via increasing the proliferation, persistence, and cytokine production of CAR-T cells in the tumor microenvironment [110].

#### Lenalidomide

Lenalidomide is a mostly utilized immunomodulatory drug that can upregulate the T cell activity by inducing the T cell proliferation, elevating the expression of kappa-light-chain-enhancer of activated B cells (NF-κB) nuclear factor, and phosphorylating the CD28 costimulatory molecule in T cells. Lenalidomide can suppress the inhibitory effect of CTLA4-Ig, a blocker of the B7-CD28 pathway, on the proliferation and cytokine production of T cells [111, 112]. Combination therapy with lenalidomide can provide a more effective immunological synapse between CAR-T cells and tumor cells expressing target antigens. Interestingly, lenalidomide reverses the functional defects in the configuration of the immunological synapse, which mostly happens in B cell malignancies like chronic lymphocytic leukemia (CLL). So, it can improve CAR-T cell function by increasing the quality of immunological synapse formation [113]. Based on this, it has been confirmed that prescribing lenalidomide with EGFRvIII-CAR-T cells elevated the F-actin polymerization and the CAR-T cell infiltration and cytotoxicity in the tumor site of the glioblastoma murine model, leading to prolonged survival of treated mice [114]. Another supporting data in Burkitt lymphoma exhibited the beneficial effects of lenalidomide accompanied by CD19-CAR-T cells in enhancing the anti-tumor cytotoxicity and IFN-γ production, which elicited an increased activity and infiltration of T cells in the tumor site and reduced tumor burden in treated mice [115]. Besides, Wang et al. reported the potent anti-tumor capability of lenalidomide combined with CD2 subset 1 (CS1)-CAR-T cell in multiple myeloma models. In this case, lenalidomide boosted the persistence and cytotoxicity of CAR-T cells, strengthened the immunological synapse formation between CAR-T cell and myeloma cells, increased the proliferation and expansion of CTLs, induced the secretion of IFN-γ and TNF-α cytokines, and inhibited the production of IL-5 and IL-10 immunosuppressive cytokines, which consequently led to successful tumor cell killing and improved survival in treated mice [116]. Based upon the pre-clinical data described above, CAR-T cell and lenalidomide co-treatment would be a rationally potent combinatorial therapeutic approach for cancers.

#### miRNAs (miR-153)

The non-coding miRNAs are epigenetic immunomodulatory molecules affecting the cellular function with different expression profiles in naive, effector, and memory T cells. In this case, miR-153 was found to act as an anti-tumor molecule in colon cancer by suppressing the Indol amine 2,3-dioxygenase 1 (IDO1) enzyme that catalyzes tryptophan kynurenine and 3-hydroxyanthranilic acid immunosuppressive metabolites. Based on this fact, combinatorial treatment of anti-EGFRvIII-CAR-T cells with overexpressed miR-153 was evaluated in xenografts model of colon cancer and demonstrated the effective tumor cell eradication [117]. IDO converts the tryptophan to inhibitory metabolites that disrupt the T cell proliferation and provide context for tumor cell escape. Therefore, it has been proved that targeting the IDO enzyme by combining the cyclophosphamide, an IDO inhibitor, with CD19 targeting CAR-T cells led to the strengthened anti-tumor cytotoxicity [118].

#### Decitabine

The density of target antigens is one of the critical parameters determining the CAR-T cell efficacy. Indeed, a lower density of target tumor antigens decreases the CAR-T cell function and frequently leads to tumor relapse after treatment, so it is essential to be regulated [119, 120]. Based on this, immunomodulators regulate the density and distribution of tumor antigens so they can overcome the heterogeneity and loss of antigens that pave the way for CAR-T cell function. Accordingly, the favorable outcome of administrating a hypomethylating component, so-called decitabine, and CAR-T cell has been confirmed in pancreatic cancer. Anurathapan et al. demonstrated that decitabine could increase the expression level of mucin 1 (MUC1) by demethylating DNA in downregulated MUC1-antigen expression and resistant CAPAN1 pancreatic tumor cells. As a result, tumor cells were predisposed to MUC1-CAR-T cells by the higher expression level of the target antigen, which led to efficient tumor cell killing [121]. Another in vitro investigation documented the positive regulatory effect of decitabine on the anti-tumor function of CD19-CAR-T cells by increasing the expression level of CD19 on lymphoma cell lines. Successfully, sequential decitabine treatment with CD19 targeting CAR-T cells in acute lymphocytic leukemia (ALL) patients indicated impressive remission with acceptable safety and efficacy [122]. Based on similar results, it has been reported that decitabine co-treatment with CD33-CAR-T cell increased the tumor cell eradication by upregulating the CD33 expression level on acute myeloid leukemia (AML) cell lines [123].

#### HDAC inhibitors (HDACis)

The histone deacetylase (HDAC) enzyme produces histone by deacetylating the acetyl group in ε-N-acetyl-lysine residue, compacting the chromatin, and suppressing the related- transcription gene [124]. In this case, utilizing the HDAC inhibitors (HDACis) increases the target gene expression on tumor cells and allows for effective cancer treatment. In Burkitt’s lymphoma, it was reported that the expression level of CD20 was upregulated on malignant B-cells after exposure to an HDACi, termed Romidepsin [125]. Interestingly, treatment with Romidepsin before CD20-CAR-T cell therapy showed a potent anti-tumor activity of CAR-T cell with increased cytotoxicity and prolonged survival of the mouse model of Burkitt’s lymphoma [126]. In AML, it has been indicated that the administration of HDAC inhibitor, namely valproic acid, prior to CAR-T cell therapy selectively upregulated the expression level of NKG2DL on low-level expressing AML cell lines and on primary AML blasts. Accordingly, valproic acid strengthened the cytotoxic, degranulation, and cytokine secretion capabilities of CAR-T cells in tumor cell eradication [127].

#### γ-secretase inhibitors (GSIs)

Plasma cells typically express the B cell maturation antigen (BCMA), a transmembrane protein, on their surface. In multiple myeloma (MM), downregulated expression levels of BSMA on myeloma cells following the CAR-T cell therapy results in relapse after about 1 month [128]. It has been shown that the multi-subunit γ-secretase complex (GS), an intra-membrane protease, reduces the CAR-T cell function via cleavage of the BCMA and subsequently secreting the soluble BCMA (sBCMA) into the circulation [129]. Applying the γ-secretase inhibitors (GSIs) to suppress the GS would help increase the CAR-T cell efficacy. From this point of view, Pont et al. investigated the combinational administration of BCMA-CAR-T cell therapy with GSIs in pre-clinical models. They reported the impressive anti-tumor function of CAR-T cells following the enhancement and reduction in BCMA and sBCMA levels, respectively, which were mediated by GSIs [130].

#### SMAC mimetics

Two main pathways mediate the tumor cell eradication and durable anti-tumor responses: releasing the cytotoxic mediators like perforin and granzymes from CTLs or CAR-T cells and cell death receptors like Fas, a member of the TNF receptor family [131]. These cell death receptors have a role in inducing tumor cell apoptosis; therefore, they contribute to the significant efficacy of NK cells, CTLs, and CAR-T cells [132]. The employment of modulatory agents to modulate the death receptor pathways can improve the anti-tumor cytotoxicity of CAR-T cells. From this point of view, the Second mitochondria-derived activator of caspase (SMAC) mimetics (SMs) have exhibited the potent efficacy in suppressing the inhibitor of apoptosis proteins (IAPs). As a result, degradation of IAPs induces the expression of TNF-α and the function of caspase 8, which subsequently elicits apoptosis in target cells [133]. It has been reported that the accompaniment of CAR-T cells and SMs would be a practical combinatorial therapeutic approach for cancers. Accordingly, three SMs have been identified as effective boosters of CD19-CAR-T cells in B-ALL [134]. As an encouraging example of synergistic combinatorial therapy, birinapant, a potent SM, increased the HER2-CAR-T cell efficacy by engaging the TNF receptor pathway, which led to successfully managing tumors in murine models [135]. Combining the CAR-T cells with SMs is another recommended therapeutic strategy that can help the better improvement of cancer cases.

### CAR-T cell combination therapy with allo-HSCT

Allogeneic hematopoietic cell transplantation (allo-HCT) and CAR-T cell therapy are considered powerful adoptive cellular therapy strategies (ACT). Encouragingly, allo-HSCT can be combined with CAR-T cells to enhance the tumor cell-killing activity and the durable remission [136]. However, complete and precise information is not available on the potentiating effect of combination therapy of CAR-T cells with allo-HSCT in cancers, which requires further research. In this context, it has been shown that CAR-T cell therapy was an effective therapeutic strategy in in relapsed/refractory (R/R) B-ALL patients with Minimal residual disease (MRD) to induce MRD-negative complete remission (CR) before allo-HSCT [137, 138].

Interestingly, it has been suggested that CAR-T cell co-treatment with allo-HSCT would decrease the leukemia relapse rate. From this point of view, several investigations support the effectiveness of this combinatorial therapy in patients who achieved the more prolonged leukemia-free survival (LFS) after receiving this treatment compared to those that received CAR-T cell monotherapy [139, 140]. Based on several clinical trials, applying consolidative allo-HSCT following the CD19 CAR-T cell therapy R/R B-ALL patients indicated the encouraging outcomes with an acceptable level of safety and efficacy. Moreover, amplified responses have been reported using the CD22 CAR-T cell therapy accompanied by allo-HSCT, which elicited proper safety and effectiveness along with Event-free survival (EFS) and overall survival (OS) [141, 142]. Safety and efficacy of allo-HSCT in adults after CD19-targeted CAR-T cell therapy was also investigated in some other B-cell malignancies such as B-cell chronic lymphocytic leukemia (B-CLL) and non-Hodgkin lymphoma (NHL) and revealed the promising results [143]. To mitigate the recurrence rate and increase the overall survival, prescribing allo-HSCT subsequent CAR-T cell therapy has been recommended in eligible high-risk patients. The importance of CAR-T cell therapy in combination with allo-HSCT has been highlighted as described above. Therefore, it should be further evaluated to determine the clinical value of this cancer treatment.

### CAR-T cell combination therapy with Metabolic inhibitors

Altering the metabolic milieu of the tumor microenvironment and modulating the metabolic profile of effector T cells in the fight against tumors are also promising therapeutic strategies, especially for solid tumors. Active T cells must be able to adapt their metabolic profile in such a way that they can act with significant proliferation, cytotoxicity, and cytokine production. However, the abnormal tumor microenvironment leads to T cell dysfunction. It is due to the higher metabolic activity of tumor cells and the creation of a hypoxic and acidic environment with a lack of essential nutrients and amino acids. On the other hand, inhibitory enzymes like arginase and IDO-1 produced by tumor cells and immunosuppressive cells cause a suppressive microenvironment by decomposing the arginine and tryptophan amino acids, respectively [144]. Therefore, targeting inhibitory enzymes, modifying the T cell metabolic profile, and enriching the required nutrients can effectively upregulate the efficacy of effector T cells and combat tumors.

Genetic modifications in T cells, such as enhancing mitochondrial function that leads to higher cytokine production by ectopic expression of PPAR-γ co-activator 1α (PCG1α), knocking out acyl-CoA cholesterol acyltransferase 1 (ACAT1) that enhances effector function of T cells, and inducing expression of catalase to resistance to hypoxia can be effective reprogramming strategies to increase the anti-tumor responses [145–147]. On the other hand, different costimulatory molecules can be used in the CAR-T cell structure to direct the cells to distinct metabolic profiles and increase cell function. For example, the presence of CD28 in CAR structure provides the aerobic glycolysis, resulting in an effector memory phenotype of CAR-T cells. However, the presence of 4-1BB increases the fatty acid oxidative breakdown and mitochondrial biogenesis, which elicits the central memory phenotype of CAR-T cells with boosted expansion and persistence [103, 148]. Synergistically, tumor cells and immunosuppressive cells [e.g., Regulatory T cells (Tregs), Tumor-associated macrophages (TAMs), Myeloid-derived suppressor cells (MDSCs), and Cancer-associated fibroblasts (CAFs)] produce inhibitory mediators like Prostaglandin E2 (PGE2), IDO, CTLA4, PD-L1, IL-10, and TGF-β as well as activate the negative signals that result in immunosuppressive TME. Thereby, altering the milieu of the tumor microenvironment would be another improvement regimen for CAR-T cell function [149]. In this case, administration of all-trans retinoic acid (ATRA) alongside CAR-T cells modulates the suppressive effects of MDSCs and improves the survival and anti-tumor responses of CARs [150]. Another recommended solution has been found that prescribing the NKG2D-based CAR-NK cells prior to CAR-T cell therapy strengthened the efficacy, persistence, and infiltration of these cells at the tumor site in a xenograft model.

Interestingly, this is followed by the significant elimination of MDSCs and tumor cells expressing the NKG2D ligand via CAR-T cells, which led to the prevention of tumor growth [151]. IDO is a critical immune-suppressive factor in the TME, causing the disability in effector T cells and increasing the expansion of immunosuppressive immune cells like Tregs, DCs, and TAMs [152]. Therefore, targeting this enzyme using IDO inhibitory drugs such as cyclophosphamide and fludarabine before CAR-T cell administration provided promising results in tumor regression and increased survival [118]. Another metabolic regulator with an inhibitory effect, Adenosine, is produced in TME and critically contributes to T cell exhaustion. The ectonucleotidases, CD39, and CD73, lead the production of Adenosine, which plays a critical role in the inhibitory function of tumor and stromal cells. Of note, targeting this metabolite alongside CAR-T cell therapy would help combat tumors [153].

## Challenges and limitations

Despite many advances in strengthening CAR-T cell function since its appearance until now, there are still many challenges regarding the efficiency and safety of these cells in the treatment of hematological and solid cancers. To combat the challenges and limit the side effects, the administration of combined therapies and the development of various innovations in the technical design of CAR-T cells can be hopeful in improving the CAR-T cell efficacy with reduced toxicity in hematological malignancies and solid cancers. Nevertheles, CAR-T cell combination therapy approaches faced by some challenges and limitations, as well. Accordingly, tumor antigen specificity and tumor antigen heterogeneity are most substantial challenges faced by CAR‑T cells and BiTEs. In this case, CAR or BiTE target antigen is expressed on normal cells in addition to tumor cells, leading to "on-target/off-tumor" toxicity. BiTEs targeting EpCAM and CAR-T cells targeting carboxy-anhydrase-IX (CA IX) on solid tumors elicit toxicity by targeting mentioned antigens in the normal gastrointestinal tract and bile duct, respectively [154, 155]. Loss of the target antigen due the tumor antigen heterogeneity is one of the most escape mechanisms for BiTEs and CAR-T cells. Lower or heterogeneous expression of target antigens on tumor cells was documented in treatments with anti-EGFRvIII- and IL13Ra2-CAR-T cell therapies and anti-CD19/CD3 BiTE (blinatumomab) [53, 156, 157].

Exposure to the treatment is associated with various challenges for CAR-T cells and BiTEs. Indeed, increased expansion and persistence of CAR-T cell therapy in hematological malignancies have resulted from lymphodepleting chemotherapy. Comparisoningly, the efficacy and expansion of CAR-T cell therapy are reduced in solid tumors. Limited expansion of anti-CEA CAR-T cells and short-time persistence of anti-HER2 CAR-T cells are examples of limited exposure to treatment by CAR-T cells [158, 159]. Besides, in BiTE therapy, the short half-life of ScFv constructs and the production of anti-drug antibodies are the main reasons for low exposure to treatment [160, 161]. Trafficking to tumor sites is another main challenge faced by BiTEs and CAR-T cells. According to studies, there is no specific trafficking to tumor sites, or cells seemed to be excluded from the center of the tumor mass. Similar difficulties may be encountered by BiTEs, which access tumor sites based on the trafficking of endogenous T cells [162].

There are still concerns and controversies about the efficacy and safety of the CAR-T cell and allo-HSCT combined treatment, and it cannot be said whether this bridge between the two treatments is useful. An antigen overlapping due to the expression of target antigens on normal progenitor- and hematopoietic stem cells remains a substantial challenge in combination therapy of CAR-T cell with allo-HSCT. Relapse and disease persistence after allo-HSCT resulting in treatment failure and death is another significant challenge in addition to the transplant-related toxicity. The reported adverse effects of allo-HSCT and CAR-T cell therapy are acute and chronic GVHD, graft failure, infections, prolonged cytopenias, neurotoxicity, and cytokine release syndrome [163, 164].

Modulating the metabolism to improve the CAR-T cell efficacy remains a significant challenge. For example, increased glycolytic metabolism promotes proliferation and cytotoxic capabilities of effector T cells but elicits poor persistence and lower differentiation of these cells into memory cells. On the other hand, increased differentiation into memory T cells but limited proliferation, migration, and cytotoxicity of CAR-T cells have resulted from the OXPHOS pathway [165]. The utilization of small molecule inhibitors in combination with CAR-T cells indicated encouraging outcomes against cancers by modulating metabolites in TME. However, these small molecules, like PI3K inhibitors, have not been entered into clinic usage for most cancers due to their lower specificity, poor stability, and extensive toxicity in vivo [166].

Lenalidomide treatment in combination with CAR-T cells has indicated improved outcomes. Nevertheless, as a serious concern, lenalidomide significantly relates to myelosuppression with grade 3 to 4 neutropenia, a well-known side effect. Also, other hematological and non-hematological toxicities, thrombopenia, anemia, infections, and thromboembolic events may occur following the administration of lenalidomide. So, this concern should be considered in using lenalidomide and CAR-T cell combination therapy [163, 167]. In another combining strategy, increased efficacy of CAR-T cells was found in combination with small molecule γ-secretase inhibitors (GSIs). By contrast, reversible inhibition of CAR-T cells was observed in administering the high concentration of GSI. On the other hand, since GSI upregulates target antigen expression on tumor cells for feasible recognition by CAR, it may elicit CAR T cell activation-induced cell death (AICD) or increased occurrence of cytokine release syndrome following the CAR T-cell administration [130].

Despite of the promising results of preclinical studies in the administration of vaccines in combination with CAR-T cells, numerous limitations still remain. Designing an efficient delivery system to lymphoid organs have been challengeable for molecular vaccine [76]. Accordingly, a vector which can be constant in circulation is needed. Cellular vaccines also showed that could be challengeable specially in the production stages. For instance; selecting proper cell line or separation of DCs from blood [168], genetic modifications on the cells for inhibiting negative immune regulators [169], loading tumor antigens on the cells [4], and finally ex-vivo expansion and activation of these cells are the main challenges for the recruiting of cellular vaccines in the greater scale. Moreover, a proper way to monitor in-vivo persistence and activation of DC-based vaccine is another challenge which has not been resolved [168].

As discussed above, researchers have indicated the positive effect of OVs as a new strategy for combination therapy with CAR-T cells, in cancer treatment. On the other hand, like any medication, clinical administration of these agents is faced with different obstacles. The most critical issue is host anti-viral immune response via neutralizing ABs and cytokine secretion, specially IFNs, which leads to viral clearance and reduced viral replication [170]. In other word, it is necessary to make balance between anti-viral and anti-tumor immune response [171]. In this regard, various strategies have been implicated such as; coating with polymers or using cellular carriers to protect OVs [172]. In addition, physical and chemical situation of TME could affect the efficacy of OVs. Cellular junction which act as a barrier for OVs penetration, as well as hypoxia and low PH which affect replication and lytic potential of some OVs are challenging for treatment [172, 173].

Despite advances made in CPB immunotherapy in cancers, some issues need to be solved. CPB as a cell-extrinsic checkpoint inhibition mechanism, encounters limitation in penetration to the tumor. Cell-intrinsic checkpoint inhibition mechanisms such as PD-1 DNR CAR-T cells and PD-1-disrupted CAR T cells can penetrate into the tumor site [174]. Due to the short-lived effects of CPM mABs the potential optimum response is dependent to multiple CPB administration, while single dose of cell-intrinsic mechanisms elicits efficient response [95].

Autoimmunity and immunotoxicity still remain challenging issues in CPB immunotherapy due to the systemic administration of CPB agents. Although CPB-induced neurotoxicity is rare, severe complication of peripheral and central nervous system have been reported, such as polyneuropathy, myositis, myasthenia gravis, demyelinating polyradiculopathy, myelitis, and encephalitis [175]. In addition, CPB-induced cardiotoxicity has been reported in more than 1% of cases, and complications such as myocarditis, pericardial disease, arrhythmia, acute coronary syndrome, and vasculitis have been reported [176]. Of note, corticosteroids have shown appropriate efficacy in managing irAEs induced by CPB [175]. Global management guidelines are required to alleviate immune-related adverse events (irAEs). Cell-intrinsic mechanisms provide localized and targeted inhibition of checkpoints activity [174]. Hence, developing cell-intrinsic mechanisms may solve CPB-induced issues and improve the efficacy.

## Concluding remarks and future directions

Management or treatment of cancers is still a serious crisis worldwide, requiring more effort to find effective treatment solutions. Today, the use of combination cancer therapies has yielded promising results. Among the various immune-based therapies, CAR-T cell therapy has been proven in many studies to be effective in multiple cancers, especially hematological ones. However, like other treatments, this method has functional limitations and doubts about efficacy and safety. Hence, numerous studies have shown that the use of CAR-T cell therapy in combination with other cancer treatments such as radiotherapy, chemotherapy, anti-cancer vaccines, oncolytic viruses, BiTEs, cytokines, checkpoint blockers, Immunomodulators, metabolic inhibitors, and allo-HSCT, have led to the synergistic effect of these therapies together. In this regard, chemotherapy and radiotherapy both cause tumor cell degradation and increase the activity of CAR-T cells and other immune cells by applying apoptosis on tumor cells and releasing DAMPs from them. On the other hand, they stimulate the production of inflammatory cytokines and inhibit the immunosuppressive cells and factors in TME. Oncolytic viruses increase the infiltration and efficacy of CAR-T cells in tumor sites through the production of RANTES, CXCL11, and IL-15. Also, they play a substantial role in DC maturation and M1 macrophage polarization. Besides, utilizing the cellular and molecular cancer vaccines is another practical strategy that helps CAR-T cells target tumor cells precisely and enhance their anti-tumor cytotoxicity. BiTEs contribute to establishing close relationships between effector cells and tumor cells by engaging two different antigens. Checkpoint inhibitors, which have recently received much attention in treating cancers, have also shown promising results in combination with CAR-T cell therapy. In this context, the use of blocking antibodies, drug antagonists, dominant-negative receptors (DNR), and knocking out mechanisms such as CRISPR/Cas9 and shRNA to suppress the inhibitory checkpoints like PD-1/PDL-1 as well as administrating the costimulatory molecules like CD28 and 4-1BB, in combination with CAR-T cell would be promised in cancer treatment. Inverted cytokine receptors (ICRs) such as 4/7 ICR and 4/21 ICR or administration of IL-2, IL-12, and IL-15 cytokines, would be other potent combination therapy with CAR-T cells. Moreover, it was claimed that exploiting the TME metabolic inhibitors would be useful. Suppressing Adenosine and IDO, increasing the mitochondrial function and cytokine production using PCG1, as well as catalase expression to counteract TME hypoxic conditions by ACAT1 are examples of TME metabolic alterations contributing to cancer improvement. Immunomodulatory factors are other anti-cancer agents that can be applied in combination with CAR-T cells, including GSIS as an inhibitor of GS, mir-155 as an IDO inhibitor, Lenidomide as an enhancer of inflammatory cytokines and inhibitor of anti-inflammatory cytokines as well as other factors such as decitabine, SMACs, HDACi, etc. Consequently, the synergistic effects of different treatments in their combined use can be more successful in degrading tumor cells and helping to regress the disease compared to monotherapy approaches.

In recent decades emerging bridging therapies, which combine various therapeutic approaches to improve the CAR-T cell efficacy, can shed light on cancer treatment. Nevertheless, there have been limited clinical data on combinatorial strategies that can be applied with CAR-T cells. More large-scale trials are required to compare the different combinatorial therapies with each other and with monotherapy. To date, there is no substantial evidence to support most of the combined treatments discussed in the current review, particularly against solid cancers. In future perspectives, augmenting CAR-T cell efficacy, increasing resist immunosuppressive TME, overcoming antigen loss and heterogeneity, metabolic reprogramming, avoiding the CAR-T cell exhaustion and immunological synapse dysfunction, improving tumor site trafficking and infiltration, and importantly restricting the CAR, adverse events should be considered as major goals for researchers' efforts in this field. The use of chemotherapy, radiotherapy, immunotherapies, targeted therapies, and other treatments in combination with CAR-T cells to reduce the tumor burden, increase the recognition and targeting of tumor antigens, maximize the stimulation of anti-tumor immune responses, and improve the survival of patients should be given special attention. Additionally, designing the next-generation CARs, such as multi-targeted CARs, programmable CARs, or armored CARs, and constructs with increased secretion of cytokines, expression of co-stimulatory molecule ligands, and suppressed tumor-derived T cell inhibitory signals, along with utilizing genome editing systems like CRISPR/Cas9 can be recommended to be paid more attention. Furthermore, designing constructs with transient CAR expression, drug-induced on/off switches, and Suicide switches mechanisms can be helpful in restricting the CAR-T cell derived toxicities. Encouragingly, multiple Clinical trials are underway or in development aiming to investigate how to improve efficacy and/or limit side effects of CAR T cells, and the world scientific community eagerly awaits their findings. There may be some possible limitations in this study that would be better discussed in future studies, including combination therapy of different types of CAR immune cells, genetic engineering and modulating approaches, and solutions for improving limitations and challenges faced by CAR-T cell combination therapies. Also, discussing more examples of preclinical and clinical studies and presenting positive and negative findings can help better understand the effectiveness of this type of combined therapy.

## Data Availability

A data availability statement is mandatory for publication in this journal.

## References

[1] Ahmad U, Khan Z, Ualiyeva D, Amissah OB, Noor Z, Khan A (2022). Chimeric antigen receptor T cell structure, its manufacturing, and related toxicities; a comprehensive review. Adva Cancer Biol-Metastasis.

[2] Elahi R, Khosh E, Tahmasebi S, Esmaeilzadeh A (2018). Immune cell hacking: challenges and clinical approaches to create smarter generations of chimeric antigen receptor T cells. Front Immunol.

[3] Zhao L, Cao YJ (2019). Engineered T cell therapy for cancer in the clinic. Front Immunol.

[4] Marofi F, Rahman HS, Al-Obaidi ZMJ, Jalil AT, Abdelbasset WK, Suksatan W (2021). Novel CAR T therapy is a ray of hope in the treatment of seriously ill AML patients. Stem Cell Res Ther.

[5] Marofi F, Tahmasebi S, Rahman HS, Kaigorodov D, Markov A, Yumashev AV (2021). Any closer to successful therapy of multiple myeloma? CAR-T cell is a good reason for optimism. Stem Cell Res Ther.

[6] Tahmasebi S, Elahi R, Esmaeilzadeh A (2019). Solid tumors challenges and new insights of CAR T cell engineering. Stem Cell Rev Rep.

[7] Marofi F, Motavalli R, Safonov VA, Thangavelu L, Yumashev AV, Alexander M (2021). CAR T cells in solid tumors: challenges and opportunities. Stem Cell Res Ther.

[8] Tahmasebi S, Elahi R, Khosh E, Esmaeilzadeh A (2021). Programmable and multi-targeted CARs: a new breakthrough in cancer CAR-T cell therapy. Clin Transl Oncol.

[9] Xu J, Wang Y, Shi J, Liu J, Li Q, Chen L (2018). Combination therapy: a feasibility strategy for CAR-T cell therapy in the treatment of solid tumors. Oncol Lett.

[10] Adkins S (2019). CAR T-cell therapy: adverse events and management. J Adv Pract Oncol.

[11] Schubert ML, Schmitt M, Wang L, Ramos CA, Jordan K, Müller-Tidow C (2021). Side-effect management of chimeric antigen receptor (CAR) T-cell therapy. Ann Oncol.

[12] Huang M, Deng J, Gao L, Zhou J (2020). Innovative strategies to advance CAR T cell therapy for solid tumors. Am J Cancer Res.

[13] Ramello MC, Haura EB, Abate-Daga D (2018). CAR-T cells and combination therapies: what’s next in the immunotherapy revolution?. Pharmacol Res.

[14] Ma Y, Adjemian S, Mattarollo SR, Yamazaki T, Aymeric L, Yang H (2013). Anticancer chemotherapy-induced intratumoral recruitment and differentiation of antigen-presenting cells. Immunity.

[15] Apetoh L, Ghiringhelli F, Tesniere A, Obeid M, Ortiz C, Criollo A (2007). Toll-like receptor 4-dependent contribution of the immune system to anticancer chemotherapy and radiotherapy. Nat Med.

[16] Garg AD, Galluzzi L, Apetoh L, Baert T, Birge RB, Bravo-San Pedro JM (2015). Molecular and translational classifications of DAMPs in immunogenic cell death. Front Immunol.

[17] Sistigu A, Yamazaki T, Vacchelli E, Chaba K, Enot DP, Adam J (2014). Cancer cell-autonomous contribution of type I interferon signaling to the efficacy of chemotherapy. Nat Med.

[18] Parente-Pereira AC, Whilding LM, Brewig N, van der Stegen SJ, Davies DM, Wilkie S (2013). Synergistic chemoimmunotherapy of epithelial ovarian cancer using ErbB-retargeted T cells combined with carboplatin. J Immunol.

[19] Heylmann D, Bauer M, Becker H, van Gool S, Bacher N, Steinbrink K (2013). Human CD4+CD25+ regulatory T cells are sensitive to low dose cyclophosphamide: implications for the immune response. PLoS ONE.

[20] Muranski P, Boni A, Wrzesinski C, Citrin DE, Rosenberg SA, Childs R (2006). Increased intensity lymphodepletion and adoptive immunotherapy–how far can we go?. Nat Clin Pract Oncol.

[21] Curran KJ, Margossian SP, Kernan NA, Silverman LB, Williams DA, Shukla N (2019). Toxicity and response after CD19-specific CAR T-cell therapy in pediatric/young adult relapsed/refractory B-ALL. Blood.

[22] Ramakrishnan R, Huang C, Cho HI, Lloyd M, Johnson J, Ren X (2012). Autophagy induced by conventional chemotherapy mediates tumor cell sensitivity to immunotherapy. Can Res.

[23] Trapani JA, Sutton VR, Thia KY, Li YQ, Froelich CJ, Jans DA (2003). A clathrin/dynamin- and mannose-6-phosphate receptor-independent pathway for granzyme B-induced cell death. J Cell Biol.

[24] Lamers CH, Willemsen R, van Elzakker P, van Steenbergen-Langeveld S, Broertjes M, Oosterwijk-Wakka J (2011). Immune responses to transgene and retroviral vector in patients treated with ex vivo-engineered T cells. Blood.

[25] Wang W, Kryczek I, Dostál L, Lin H, Tan L, Zhao L (2016). Effector T cells abrogate stroma-mediated chemoresistance in ovarian cancer. Cell.

[26] Michaud M, Martins I, Sukkurwala AQ, Adjemian S, Ma Y, Pellegatti P (2011). Autophagy-dependent anticancer immune responses induced by chemotherapeutic agents in mice. Science.

[27] Dangaj D, Bruand M, Grimm AJ, Ronet C, Barras D, Duttagupta PA (2019). Cooperation between constitutive and inducible chemokines enables T cell engraftment and immune attack in solid tumors. Cancer Cell.

[28] Pfirschke C, Engblom C, Rickelt S, Cortez-Retamozo V, Garris C, Pucci F (2016). Immunogenic chemotherapy sensitizes tumors to checkpoint blockade therapy. Immunity.

[29] Paulsson J, Micke P (2014). Prognostic relevance of cancer-associated fibroblasts in human cancer. Semin Cancer Biol.

[30] Gardner RA, Finney O, Annesley C, Brakke H, Summers C, Leger K (2017). Intent-to-treat leukemia remission by CD19 CAR T cells of defined formulation and dose in children and young adults. Blood.

[31] Porter DL, Hwang WT, Frey NV, Lacey SF, Shaw PA, Loren AW (2015). Chimeric antigen receptor T cells persist and induce sustained remissions in relapsed refractory chronic lymphocytic leukemia. Sci Transl Med.

[32] Shevtsov M, Sato H, Multhoff G, Shibata A (2019). Novel approaches to improve the efficacy of immuno-radiotherapy. Front Oncol.

[33] Lee Y, Auh SL, Wang Y, Burnette B, Wang Y, Meng Y (2009). Therapeutic effects of ablative radiation on local tumor require CD8+ T cells: changing strategies for cancer treatment. Blood.

[34] Minn I, Rowe SP, Pomper MG (2019). Enhancing CAR T-cell therapy through cellular imaging and radiotherapy. Lancet Oncol.

[35] Reits EA, Hodge JW, Herberts CA, Groothuis TA, Chakraborty M, Wansley EK (2006). Radiation modulates the peptide repertoire, enhances MHC class I expression, and induces successful antitumor immunotherapy. J Exp Med.

[36] Higgins JP, Bernstein MB, Hodge JW (2009). Enhancing immune responses to tumor-associated antigens. Cancer Biol Ther.

[37] Lugade AA, Sorensen EW, Gerber SA, Moran JP, Frelinger JG, Lord EM (2008). Radiation-induced IFN-gamma production within the tumor microenvironment influences antitumor immunity. J Immunol.

[38] Zhang B, Bowerman NA, Salama JK, Schmidt H, Spiotto MT, Schietinger A (2007). Induced sensitization of tumor stroma leads to eradication of established cancer by T cells. J Exp Med.

[39] Demaria S, Ng B, Devitt ML, Babb JS, Kawashima N, Liebes L (2004). Ionizing radiation inhibition of distant untreated tumors (abscopal effect) is immune mediated. Int J Radiat Oncol Biol Phys.

[40] Weiss T, Weller M, Guckenberger M, Sentman CL, Roth P (2018). NKG2D-based CAR T cells and radiotherapy exert synergistic efficacy in glioblastoma. Can Res.

[41] DeSelm C, Palomba ML, Yahalom J, Hamieh M, Eyquem J, Rajasekhar VK (2018). Low-dose radiation conditioning enables CAR T cells to mitigate antigen escape. Mol Ther.

[42] Gaipl US, Multhoff G, Scheithauer H, Lauber K, Hehlgans S, Frey B (2014). Kill and spread the word: stimulation of antitumor immune responses in the context of radiotherapy. Immunotherapy.

[43] Russell SJ, Peng KW, Bell JC (2012). Oncolytic virotherapy. Nat Biotechnol.

[44] Zhang Y, Li Y, Chen K, Qian L, Wang P (2021). Oncolytic virotherapy reverses the immunosuppressive tumor microenvironment and its potential in combination with immunotherapy. Cancer Cell Int.

[45] Guedan S, Alemany R (2018). CAR-T cells and oncolytic viruses: joining forces to overcome the solid tumor challenge. Front Immunol.

[46] Nishio N, Diaconu I, Liu H, Cerullo V, Caruana I, Hoyos V (2014). Armed oncolytic virus enhances immune functions of chimeric antigen receptor-modified T cells in solid tumors. Can Res.

[47] Moon EK, Wang LS, Bekdache K, Lynn RC, Lo A, Thorne SH (2018). Intra-tumoral delivery of CXCL11 via a vaccinia virus, but not by modified T cells, enhances the efficacy of adoptive T cell therapy and vaccines. Oncoimmunology.

[48] Watanabe K, Luo Y, Da T, Guedan S, Ruella M, Scholler J (2018). Pancreatic cancer therapy with combined mesothelin-redirected chimeric antigen receptor T cells and cytokine-armed oncolytic adenoviruses. JCI Insight.

[49] Li Y, Xiao F, Zhang A, Zhang D, Nie W, Xu T (2020). Oncolytic adenovirus targeting TGF-β enhances anti-tumor responses of mesothelin-targeted chimeric antigen receptor T cell therapy against breast cancer. Cell Immunol.

[50] Day D, Hansen AR (2016). Immune-related adverse events associated with immune checkpoint inhibitors. BioDrugs.

[51] Tanoue K, Rosewell Shaw A, Watanabe N, Porter C, Rana B, Gottschalk S (2017). Armed oncolytic adenovirus-expressing PD-L1 mini-body enhances antitumor effects of chimeric antigen receptor T cells in solid tumors. Can Res.

[52] Rosewell Shaw A, Porter CE, Watanabe N, Tanoue K, Sikora A, Gottschalk S (2017). Adenovirotherapy delivering cytokine and checkpoint inhibitor augments CAR T cells against metastatic head and neck cancer. Mol Ther.

[53] O'Rourke DM, Nasrallah MP, Desai A, Melenhorst JJ, Mansfield K, Morrissette JJD (2017). A single dose of peripherally infused EGFRvIII-directed CAR T cells mediates antigen loss and induces adaptive resistance in patients with recurrent glioblastoma. Sci Transl Med.

[54] Schmidts A, Maus MV (2018). Making CAR T cells a solid option for solid tumors. Front Immunol.

[55] Park AK, Fong Y, Kim SI, Yang J, Murad JP, Lu J (2020). Effective combination immunotherapy using oncolytic viruses to deliver CAR targets to solid tumors. Sci Transl Med.

[56] Wing A, Fajardo CA, Posey AD, Shaw C, Da T, Young RM (2018). Improving CART-cell therapy of solid tumors with oncolytic virus-driven production of a bispecific T-cell engager. Cancer Immunol Res.

[57] Porter CE, Rosewell Shaw A, Jung Y, Yip T, Castro PD, Sandulache VC (2020). Oncolytic adenovirus armed with BiTE, cytokine, and checkpoint inhibitor enables CAR T cells to control the growth of heterogeneous tumors. Mol Ther.

[58] Wang X, Gottschalk S, Song X-T (2014). Synergistic antitumor effects of Chimeric antigen receptor-modified T cells and Oncolytic Virotherapy.

[59] VanSeggelen H, Tantalo DG, Afsahi A, Hammill JA, Bramson JL (2015). Chimeric antigen receptor-engineered T cells as oncolytic virus carriers. Mol Ther Oncol.

[60] Bansal R, Reshef R (2021). Revving the CAR—combination strategies to enhance CAR T cell effectiveness. Blood Rev.

[61] Avigan D, Rosenblatt J (2018). Vaccine therapy in hematologic malignancies. Blood.

[62] Le DT, Pardoll DM, Jaffee EM (2010). Cellular vaccine approaches. Cancer J.

[63] Caruana I, Weber G, Ballard BC, Wood MS, Savoldo B, Dotti G (2015). K562-derived whole-cell vaccine enhances antitumor responses of CAR-redirected virus-specific cytotoxic T lymphocytes in vivo. Clin Cancer Res.

[64] Rossig C, Pule M, Altvater B, Saiagh S, Wright G, Ghorashian S (2017). Vaccination to improve the persistence of CD19CAR gene-modified T cells in relapsed pediatric acute lymphoblastic leukemia. Leukemia.

[65] Weinstock M, Rosenblatt J, Avigan D (2017). Dendritic cell therapies for hematologic malignancies. Mol Ther.

[66] Wu M, Zhang L, Zhang H, Ning J, Tu S, He Y (2019). CD19 chimeric antigen receptor-redirected T cells combined with epidermal growth factor receptor pathway substrate 8 peptide-derived dendritic cell vaccine in leukemia. Cytotherapy.

[67] Chan JD, von Scheidt B, Zeng B, Oliver AJ, Davey AS, Ali AI (2020). Enhancing chimeric antigen receptor T-cell immunotherapy against cancer using a nanoemulsion-based vaccine targeting cross-presenting dendritic cells. Clin Transl Immunol.

[68] Reinhard K, Rengstl B, Oehm P, Michel K, Billmeier A, Hayduk N (2020). An RNA vaccine drives expansion and efficacy of claudin-CAR-T cells against solid tumors. Science.

[69] Beck JD, Reidenbach D, Salomon N, Sahin U, Türeci Ö, Vormehr M (2021). mRNA therapeutics in cancer immunotherapy. Mol Cancer.

[70] Cooper LJ, Al-Kadhimi Z, Serrano LM, Pfeiffer T, Olivares S, Castro A (2005). Enhanced antilymphoma efficacy of CD19-redirected influenza MP1-specific CTLs by cotransfer of T cells modified to present influenza MP1. Blood.

[71] Cruz CR, Micklethwaite KP, Savoldo B, Ramos CA, Lam S, Ku S (2013). Infusion of donor-derived CD19-redirected virus-specific T cells for B-cell malignancies relapsed after allogeneic stem cell transplant: a phase 1 study. Blood.

[72] Tanaka M, Tashiro H, Omer B, Lapteva N, Ando J, Ngo M (2017). Vaccination targeting native receptors to enhance the function and proliferation of chimeric antigen receptor (CAR)-modified T cells. Clin Cancer Res.

[73] Wang X, Wong CW, Urak R, Mardiros A, Budde LE, Chang WC (2015). CMVpp65 vaccine enhances the antitumor efficacy of adoptively transferred CD19-redirected CMV-specific T cells. Clin Cancer Res.

[74] Bansal R, Reshef R (2021). Revving the CAR–Combination strategies to enhance CAR T cell effectiveness. Blood Rev.

[75] Akahori Y, Wang L, Yoneyama M, Seo N, Okumura S, Miyahara Y (2018). Antitumor activity of CAR-T cells targeting the intracellular oncoprotein WT1 can be enhanced by vaccination. Blood.

[76] Liu H, Moynihan KD, Zheng Y, Szeto GL, Li AV, Huang B (2014). Structure-based programming of lymph-node targeting in molecular vaccines. Nature.

[77] Slaney CY, von Scheidt B, Davenport AJ, Beavis PA, Westwood JA, Mardiana S (2017). Dual-specific chimeric antigen receptor T cells and an indirect vaccine eradicate a variety of large solid tumors in an immunocompetent, self-antigen setting. Clin Cancer Res.

[78] Mohammed S, Sukumaran S, Bajgain P, Watanabe N, Heslop HE, Rooney CM (2017). Improving chimeric antigen receptor-modified T cell function by reversing the immunosuppressive tumor microenvironment of pancreatic cancer. Mol Ther.

[79] Wang Y, Jiang H, Luo H, Sun Y, Shi B, Sun R (2019). An IL-4/21 inverted cytokine receptor improving CAR-T cell potency in immunosuppressive solid-tumor microenvironment. Front Immunol.

[80] Fraietta JA, Lacey SF, Orlando EJ, Pruteanu-Malinici I, Gohil M, Lundh S (2018). Determinants of response and resistance to CD19 chimeric antigen receptor (CAR) T cell therapy of chronic lymphocytic leukemia. Nat Med.

[81] Perna SK, Pagliara D, Mahendravada A, Liu H, Brenner MK, Savoldo B (2014). Interleukin-7 mediates selective expansion of tumor-redirected cytotoxic T lymphocytes (CTLs) without enhancement of regulatory T-cell inhibition. Clinical cancer Research.

[82] Wei J, Sun H, Zhang A, Wu X, Li Y, Liu J (2018). A novel AXL chimeric antigen receptor endows T cells with anti-tumor effects against triple negative breast cancers. Cell Immunol.

[83] Zhao Z, Li Y, Liu W, Li X (2020). Engineered IL-7 receptor enhances the therapeutic effect of AXL-CAR-T cells on triple-negative breast cancer. Biomed Res Int.

[84] Hsu C, Hughes MS, Zheng Z, Bray RB, Rosenberg SA, Morgan RA (2005). Primary human T lymphocytes engineered with a codon-optimized IL-15 gene resist cytokine withdrawal-induced apoptosis and persist long-term in the absence of exogenous cytokine. J Immunol.

[85] Hoyos V, Savoldo B, Quintarelli C, Mahendravada A, Zhang M, Vera J (2010). Engineering CD19-specific T lymphocytes with interleukin-15 and a suicide gene to enhance their anti-lymphoma/leukemia effects and safety. Leukemia.

[86] Alizadeh D, Wong RA, Yang X, Wang D, Pecoraro JR, Kuo CF (2019). IL15 Enhances CAR-T cell antitumor activity by reducing mTORC1 activity and preserving their stem cell memory phenotype. Cancer Immunol Res.

[87] Lanitis E, Rota G, Kosti P, Ronet C, Spill A, Seijo B (2021). Optimized gene engineering of murine CAR-T cells reveals the beneficial effects of IL-15 coexpression. J Exp Med.

[88] Battram AM, Bachiller M, Lopez V, Fernández de Larrea C, Urbano-Ispizua A, Martín-Antonio B (2021). IL-15 enzhances the persistence and function of BCMA-targeting CAR-T cells compared to IL-2 or IL-15/IL-7 by limiting CAR-T cell dysfunction and differentiation. Cancers.

[89] Hurton LV, Singh H, Najjar AM, Switzer KC, Mi T, Maiti S (2016). Tethered IL-15 augments antitumor activity and promotes a stem-cell memory subset in tumor-specific T cells. Proc Natl Acad Sci USA.

[90] Gargett T, Yu W, Dotti G, Yvon ES, Christo SN, Hayball JD (2016). GD2-specific CAR T cells undergo potent activation and deletion following antigen encounter but can be protected from activation-induced cell death by PD-1 blockade. Mol Ther.

[91] John LB, Devaud C, Duong CP, Yong CS, Beavis PA, Haynes NM (2013). Anti-PD-1 antibody therapy potently enhances the eradication of established tumors by gene-modified T cells. Clin Cancer Res.

[92] Hofmann L, Forschner A, Loquai C, Goldinger SM, Zimmer L, Ugurel S (2016). Cutaneous, gastrointestinal, hepatic, endocrine, and renal side-effects of anti-PD-1 therapy. Eur J Cancer.

[93] Shivaji UN, Jeffery L, Gui X, Smith SCL, Ahmad OF, Akbar A (2019). Immune checkpoint inhibitor-associated gastrointestinal and hepatic adverse events and their management. Ther Adv Gastroenterol.

[94] Giuffrida L, Sek K, Henderson MA, House IG, Lai J, Chen AXY (2020). IL-15 preconditioning augments CAR T cell responses to checkpoint blockade for improved treatment of solid tumors. Mol Ther.

[95] Cherkassky L, Morello A, Villena-Vargas J, Feng Y, Dimitrov DS, Jones DR (2016). Human CAR T cells with cell-intrinsic PD-1 checkpoint blockade resist tumor-mediated inhibition. J Clin Investig.

[96] Beavis PA, Henderson MA, Giuffrida L, Mills JK, Sek K, Cross RS (2017). Targeting the adenosine 2A receptor enhances chimeric antigen receptor T cell efficacy. J Clin Investig.

[97] Condomines M, Arnason J, Benjamin R, Gunset G, Plotkin J, Sadelain M (2015). Tumor-targeted human T cells expressing CD28-based chimeric antigen receptors circumvent CTLA-4 inhibition. PLoS ONE.

[98] Hu W, Zi Z, Jin Y, Li G, Shao K, Cai Q (2019). CRISPR/Cas9-mediated PD-1 disruption enhances human mesothelin-targeted CAR T cell effector functions. Cancer Immunol Immunother.

[99] Rupp LJ, Schumann K, Roybal KT, Gate RE, Ye CJ, Lim WA (2017). CRISPR/Cas9-mediated PD-1 disruption enhances anti-tumor efficacy of human chimeric antigen receptor T cells. Sci Rep.

[100] Zhang Y, Zhang X, Cheng C, Mu W, Liu X, Li N (2017). CRISPR-Cas9 mediated LAG-3 disruption in CAR-T cells. Front Med.

[101] Choi BD, Yu X, Castano AP, Darr H, Henderson DB, Bouffard AA (2019). CRISPR-Cas9 disruption of PD-1 enhances activity of universal EGFRvIII CAR T cells in a preclinical model of human glioblastoma. J Immunother Cancer.

[102] Ren J, Zhang X, Liu X, Fang C, Jiang S, June CH (2017). A versatile system for rapid multiplex genome-edited CAR T cell generation. Oncotarget.

[103] Long AH, Haso WM, Shern JF, Wanhainen KM, Murgai M, Ingaramo M (2015). 4–1BB costimulation ameliorates T cell exhaustion induced by tonic signaling of chimeric antigen receptors. Nat Med.

[104] Suurs FV, Lub-de Hooge MN, de Vries EGE, de Groot DJA (2019). A review of bispecific antibodies and antibody constructs in oncology and clinical challenges. Pharmacol Ther.

[105] Klinger M, Benjamin J, Kischel R, Stienen S, Zugmaier G (2016). Harnessing T cells to fight cancer with BiTE^®^ antibody constructs–past developments and future directions. Immunol Rev.

[106] Choi BD, Yu X, Castano AP, Bouffard AA, Schmidts A, Larson RC (2019). CAR-T cells secreting BiTEs circumvent antigen escape without detectable toxicity. Nat Biotechnol.

[107] Shalabi H, Koegel A, Ponduri A, Qin H, Salem D, Stetler-Stevenson M (2019). Case report: impact of BITE on CAR-T cell expansion. Adva Cell Gene Ther.

[108] Kim MS, Ma JS, Yun H, Cao Y, Kim JY, Chi V (2015). Redirection of genetically engineered CAR-T cells using bifunctional small molecules. J Am Chem Soc.

[109] Tamada K, Geng D, Sakoda Y, Bansal N, Srivastava R, Li Z (2012). Redirecting gene-modified T cells toward various cancer types using tagged antibodies. Clin Cancer Res.

[110] Schafer PH, Gandhi AK, Loveland MA, Chen RS, Man HW, Schnetkamp PP (2003). Enhancement of cytokine production and AP-1 transcriptional activity in T cells by thalidomide-related immunomodulatory drugs. J Pharmacol Exp Ther.

[111] Kim BS, Kim JY, Kim EJ, Lee JG, Joo DJ, Huh KH (2016). Role of thalidomide on the expression of OX40, 4–1BB, and GITR in T cell subsets. Transpl Proc.

[112] LeBlanc R, Hideshima T, Catley LP, Shringarpure R, Burger R, Mitsiades N (2004). Immunomodulatory drug costimulates T cells via the B7-CD28 pathway. Blood.

[113] Ramsay AG, Johnson AJ, Lee AM, Gorgün G, Le Dieu R, Blum W (2008). Chronic lymphocytic leukemia T cells show impaired immunological synapse formation that can be reversed with an immunomodulating drug. J Clin Investig.

[114] Kuramitsu S, Ohno M, Ohka F, Shiina S, Yamamichi A, Kato A (2015). Lenalidomide enhances the function of chimeric antigen receptor T cells against the epidermal growth factor receptor variant III by enhancing immune synapses. Cancer Gene Ther.

[115] Otáhal P, Průková D, Král V, Fabry M, Vočková P, Latečková L (2016). Lenalidomide enhances antitumor functions of chimeric antigen receptor modified T cells. Oncoimmunology.

[116] Wang X, Walter M, Urak R, Weng L, Huynh C, Lim L (2018). Lenalidomide enhances the function of CS1 chimeric antigen receptor-redirected T cells against multiple myeloma. Clin Cancer Res.

[117] Huang Q, Xia J, Wang L, Wang X, Ma X, Deng Q (2018). miR-153 suppresses IDO1 expression and enhances CAR T cell immunotherapy. J Hematol Oncol.

[118] Ninomiya S, Narala N, Huye L, Yagyu S, Savoldo B, Dotti G (2015). Tumor indoleamine 2,3-dioxygenase (IDO) inhibits CD19-CAR T cells and is downregulated by lymphodepleting drugs. Blood.

[119] Fry TJ, Shah NN, Orentas RJ, Stetler-Stevenson M, Yuan CM, Ramakrishna S (2018). CD22-targeted CAR T cells induce remission in B-ALL that is naive or resistant to CD19-targeted CAR immunotherapy. Nat Med.

[120] Watanabe K, Terakura S, Martens AC, van Meerten T, Uchiyama S, Imai M (2015). Target antigen density governs the efficacy of anti-CD20-CD28-CD3 ζ chimeric antigen receptor-modified effector CD8+ T cells. J Immunol.

[121] Anurathapan U, Chan RC, Hindi HF, Mucharla R, Bajgain P, Hayes BC (2014). Kinetics of tumor destruction by chimeric antigen receptor-modified T cells. Mol Ther.

[122] Li S, Xue L, Wang M, Qiang P, Xu H, Zhang X (2019). Decitabine enhances cytotoxic effect of T cells with an anti-CD19 chimeric antigen receptor in treatment of lymphoma. Onco Targets Ther.

[123] Xue T, Del Real M, Marcucci E, Toribio C, Setayesh SM, Forman SJ (2019). Checkpoint blockade in combination with CD33 chimeric antigen receptor T cell therapy and hypomethylating agent against acute myeloid leukemia. Blood.

[124] Bradner JE, West N, Grachan ML, Greenberg EF, Haggarty SJ, Warnow T (2010). Chemical phylogenetics of histone deacetylases. Nat Chem Biol.

[125] Chu Y, Hochberg J, Yahr A, Ayello J, van de Ven C, Barth M (2015). Targeting CD20+ aggressive B-cell non-hodgkin lymphoma by Anti-CD20 CAR mRNA-modified expanded natural killer cells in vitro and in NSG mice. Cancer Immunol Res.

[126] Xu Y, Li S, Wang Y, Liu J, Mao X, Xing H (2019). Induced CD20 expression on B-cell malignant cells heightened the cytotoxic activity of chimeric antigen receptor engineered T cells. Hum Gene Ther.

[127] Driouk L, Gicobi JK, Kamihara Y, Rutherford K, Dranoff G, Ritz J (2020). Chimeric antigen receptor T cells targeting NKG2D-ligands show robust efficacy against acute myeloid leukemia and T-cell acute lymphoblastic leukemia. Front Immunol.

[128] Brudno JN, Maric I, Hartman SD, Rose JJ, Wang M, Lam N (2018). T Cells genetically modified to express an Anti-B-cell maturation antigen chimeric antigen receptor cause remissions of poor-prognosis relapsed multiple myeloma. J Clin Oncol.

[129] Laurent SA, Hoffmann FS, Kuhn PH, Cheng Q, Chu Y, Schmidt-Supprian M (2015). γ-Secretase directly sheds the survival receptor BCMA from plasma cells. Nat Commun.

[130] Pont MJ, Hill T, Cole GO, Abbott JJ, Kelliher J, Salter AI (2019). γ-Secretase inhibition increases efficacy of BCMA-specific chimeric antigen receptor T cells in multiple myeloma. Blood.

[131] Benmebarek MR, Karches CH, Cadilha BL, Lesch S, Endres S, Kobold S (2019). Killing mechanisms of chimeric antigen receptor (CAR) T cells. Int J Mol Sci.

[132] Kearney CJ, Vervoort SJ, Hogg SJ, Ramsbottom KM, Freeman AJ, Lalaoui N (2018). Tumor immune evasion arises through loss of TNF sensitivity. Sci Immunol.

[133] Petersen SL, Wang L, Yalcin-Chin A, Li L, Peyton M, Minna J (2007). Autocrine TNFalpha signaling renders human cancer cells susceptible to Smac-mimetic-induced apoptosis. Cancer Cell.

[134] Dufva O, Koski J, Maliniemi P, Ianevski A, Klievink J, Leitner J (2020). Integrated drug profiling and CRISPR screening identify essential pathways for CAR T-cell cytotoxicity. Blood.

[135] Michie J, Beavis PA, Freeman AJ, Vervoort SJ, Ramsbottom KM, Narasimhan V (2019). Antagonism of IAPs enhances CAR T-cell efficacy. Cancer Immunol Res.

[136] Zhao XS, Liu YR, Xu LP, Wang Y, Zhang XH, Chen H (2019). Minimal residual disease status determined by multiparametric flow cytometry pretransplantation predicts the outcome of patients with ALL receiving unmanipulated haploidentical allografts. Am J Hematol.

[137] Khazal S, Kebriaei P (2020). Debate: transplant is still necessary in the era of targeted cellular therapy for acute lymphoblastic leukemia. Clin Lymphoma Myeloma Leuk.

[138] Taraseviciute A, Broglie L, Phelan R, Bhatt NS, Becktell K, Burke MJ (2019). What is the role of hematopoietic cell transplantation (HCT) for pediatric acute lymphoblastic leukemia (ALL) in the age of chimeric antigen receptor T-cell (CART) therapy?. J Pediatr Hematol Oncol.

[139] Hay KA, Gauthier J, Hirayama AV, Voutsinas JM, Wu Q, Li D (2019). Factors associated with durable EFS in adult B-cell ALL patients achieving MRD-negative CR after CD19 CAR T-cell therapy. Blood.

[140] Jiang H, Li C, Yin P, Guo T, Liu L, Xia L (2019). Anti-CD19 chimeric antigen receptor-modified T-cell therapy bridging to allogeneic hematopoietic stem cell transplantation for relapsed/refractory B-cell acute lymphoblastic leukemia: an open-label pragmatic clinical trial. Am J Hematol.

[141] Jiang H, Hu Y, Mei H (2020). Consolidative allogeneic hematopoietic stem cell transplantation after chimeric antigen receptor T-cell therapy for relapsed/refractory B-cell acute lymphoblastic leukemia: who? When? Why?. Biomarker Res.

[142] Li L, Liu J, Xu M, Yu H, Lv C, Cao F (2020). Treatment response, survival, safety, and predictive factors to chimeric antigen receptor T cell therapy in Chinese relapsed or refractory B cell acute lymphoblast leukemia patients. Cell Death Dis.

[143] Shadman M, Gauthier J, Hay KA, Voutsinas JM, Milano F, Li A (2019). Safety of allogeneic hematopoietic cell transplant in adults after CD19-targeted CAR T-cell therapy. Blood Adv.

[144] Irving M, Vuillefroy de Silly R, Scholten K, Dilek N, Coukos G (2017). Engineering chimeric antigen receptor T-cells for racing in solid tumors: don't forget the fuel. Front Immunol.

[145] Ligtenberg MA, Mougiakakos D, Mukhopadhyay M, Witt K, Lladser A, Chmielewski M (2016). Coexpressed catalase protects chimeric antigen receptor-redirected T cells as well as bystander cells from oxidative stress-induced loss of antitumor activity. J Immunol.

[146] Scharping NE, Menk AV, Moreci RS, Whetstone RD, Dadey RE, Watkins SC (2016). The tumor microenvironment represses T cell mitochondrial biogenesis to drive intratumoral T cell metabolic insufficiency and dysfunction. Immunity.

[147] Yang W, Bai Y, Xiong Y, Zhang J, Chen S, Zheng X (2016). Potentiating the antitumour response of CD8(+) T cells by modulating cholesterol metabolism. Nature.

[148] Kawalekar OU, O'Connor RS, Fraietta JA, Guo L, McGettigan SE, Posey AD (2016). Distinct signaling of coreceptors regulates specific metabolism pathways and impacts memory development in CAR T cells. Immunity.

[149] Rabinovich GA, Gabrilovich D, Sotomayor EM (2007). Immunosuppressive strategies that are mediated by tumor cells. Annu Rev Immunol.

[150] Long AH, Highfill SL, Cui Y, Smith JP, Walker AJ, Ramakrishna S (2016). Reduction of MDSCs with All-trans retinoic acid improves CAR therapy efficacy for sarcomas. Cancer Immunol Res.

[151] Parihar R, Rivas C, Huynh M, Omer B, Lapteva N, Metelitsa LS (2019). NK cells expressing a chimeric activating receptor eliminate MDSCs and rescue Impaired CAR-T cell activity against solid tumors. Cancer Immunol Res.

[152] Mezrich JD, Fechner JH, Zhang X, Johnson BP, Burlingham WJ, Bradfield CA (2010). An interaction between kynurenine and the aryl hydrocarbon receptor can generate regulatory T cells. J Immunol.

[153] Allard B, Longhi MS, Robson SC, Stagg J (2017). The ectonucleotidases CD39 and CD73: Novel checkpoint inhibitor targets. Immunol Rev.

[154] Kebenko M, Goebeler ME, Wolf M, Hasenburg A, Seggewiss-Bernhardt R, Ritter B (2018). A multicenter phase 1 study of solitomab (MT110, AMG 110), a bispecific EpCAM/CD3 T-cell engager (BiTE^®^) antibody construct, in patients with refractory solid tumors. Oncoimmunology.

[155] Lamers CH, Klaver Y, Gratama JW, Sleijfer S, Debets R (2016). Treatment of metastatic renal cell carcinoma (mRCC) with CAIX CAR-engineered T-cells-a completed study overview. Biochem Soc Trans.

[156] Braig F, Brandt A, Goebeler M, Tony HP, Kurze AK, Nollau P (2017). Resistance to anti-CD19/CD3 BiTE in acute lymphoblastic leukemia may be mediated by disrupted CD19 membrane trafficking. Blood.

[157] Brown CE, Badie B, Barish ME, Weng L, Ostberg JR, Chang WC (2015). Bioactivity and safety of IL13Rα2-redirected chimeric antigen receptor CD8+ T cells in patients with recurrent glioblastoma. Clin Cancer Res.

[158] Ahmed N, Brawley VS, Hegde M, Robertson C, Ghazi A, Gerken C (2015). Human epidermal growth factor receptor 2 (HER2) -specific chimeric antigen receptor-modified T cells for the Immunotherapy of HER2-positive sarcoma. J Clin Oncol.

[159] Thistlethwaite FC, Gilham DE, Guest RD, Rothwell DG, Pillai M, Burt DJ (2017). The clinical efficacy of first-generation carcinoembryonic antigen (CEACAM5)-specific CAR T cells is limited by poor persistence and transient pre-conditioning-dependent respiratory toxicity. Cancer Immunol Immunother.

[160] Moek K, Fiedler W, von Einem J, Verheul H, Seufferlein T, de Groot D (2018). Phase I study of AMG 211/MEDI-565 administered as continuous intravenous infusion (cIV) for relapsed/refractory gastrointestinal (GI) adenocarcinoma. Ann Oncol.

[161] Pishvaian M, Morse MA, McDevitt J, Norton JD, Ren S, Robbie GJ (2016). Phase 1 dose escalation study of MEDI-565, a bispecific T-Cell engager that targets human carcinoembryonic antigen, in patients with advanced gastrointestinal adenocarcinomas. Clin Colorectal Cancer.

[162] Edeline J, Houot R, Marabelle A, Alcantara M (2021). CAR-T cells and BiTEs in solid tumors: challenges and perspectives. J Hematol Oncol.

[163] Bartoló-Ibars A, Uribe-Herranz M, Muñoz-Sánchez G, Arnaldos-Pérez C, Ortiz-Maldonado V, Urbano-Ispizua Á (2021). CAR-T after stem cell transplantation in B-cell lymphoproliferative disorders: are they really autologous or allogenic cell therapies?. Cancers..

[164] Goldsmith SR, Ghobadi A, DiPersio JF (2020). Hematopoeitic cell transplantation and CAR T-cell therapy: complements or competitors?. Front Oncol.

[165] Zhang M, Jin X, Sun R, Xiong X, Wang J, Xie D (2021). Optimization of metabolism to improve efficacy during CAR-T cell manufacturing. J Transl Med.

[166] Yang J, Nie J, Ma X, Wei Y, Peng Y, Wei X (2019). Targeting PI3K in cancer: mechanisms and advances in clinical trials. Mol Cancer.

[167] Leleu X, Terpos E, Sanz RG, Cooney J, O'Gorman P, Minarik J (2016). An international, multicenter, prospective, observational study of neutropenia in patients being treated with lenalidomide + dexamethasone for relapsed or relapsed/refractory multiple myeloma (RR-MM). Am J Hematol.

[168] Abakushina EV, Popova LI, Zamyatnin AA, Werner J, Mikhailovsky NV, Bazhin AV (2021). The advantages and challenges of anticancer dendritic cell vaccines and NK cells in adoptive cell immunotherapy. Vaccines.

[169] Ramachandran M, Dimberg A, Essand M (2017). The cancer-immunity cycle as rational design for synthetic cancer drugs: novel DC vaccines and CAR T-cells. Semin Cancer Biol.

[170] Lauer UM, Beil J (2022). Oncolytic viruses: challenges and considerations in an evolving clinical landscape. Future Oncol.

[171] Zheng M, Huang J, Tong A, Yang H (2019). Oncolytic viruses for cancer therapy: barriers and recent advances. Mol Ther Oncol.

[172] Goradel NH, Baker AT, Arashkia A, Ebrahimi N, Ghorghanlu S, Negahdari B (2021). Oncolytic virotherapy: challenges and solutions. Curr Probl Cancer.

[173] Jin KT, Du WL, Liu YY, Lan HR, Si JX, Mou XZ (2021). Oncolytic virotherapy in solid tumors: the challenges and achievements. Cancers.

[174] Grosser R, Cherkassky L, Chintala N, Adusumilli PS (2019). Combination immunotherapy with CAR T cells and checkpoint blockade for the treatment of solid tumors. Cancer Cell.

[175] Perrinjaquet C, Desbaillets N, Hottinger AF (2019). Neurotoxicity associated with cancer immunotherapy: immune checkpoint inhibitors and chimeric antigen receptor T-cell therapy. Curr Opin Neurol.

[176] Stein-Merlob AF, Rothberg MV, Holman P, Yang EH (2021). Immunotherapy-associated cardiotoxicity of immune checkpoint inhibitors and chimeric antigen receptor T cell therapy: diagnostic and management challenges and strategies. Curr Cardiol Rep.

